# A decision-theoretic approach to Bayesian clinical trial design and evaluation of robustness to prior-data conflict

**DOI:** 10.1093/biostatistics/kxaa027

**Published:** 2020-07-31

**Authors:** Silvia Calderazzo, Manuel Wiesenfarth, Annette Kopp-Schneider

**Affiliations:** Division of Biostatistics, German Cancer Research Center, Im Neuenheimer Feld 581, 69120 Heidelberg, Germany

**Keywords:** Average type I error rate, Bayesian clinical trial design, Bayesian decision theory, Prior-data conflict, Robust prior, Sampling-analysis prior

## Abstract

Bayesian clinical trials allow taking advantage of relevant external information through the elicitation of prior distributions, which influence Bayesian posterior parameter estimates and test decisions. However, incorporation of historical information can have harmful consequences on the trial’s frequentist (conditional) operating characteristics in case of inconsistency between prior information and the newly collected data. A compromise between meaningful incorporation of historical information and strict control of frequentist error rates is therefore often sought. Our aim is thus to review and investigate the rationale and consequences of different approaches to relaxing strict frequentist control of error rates from a Bayesian decision-theoretic viewpoint. In particular, we define an integrated risk which incorporates losses arising from testing, estimation, and sampling. A weighted combination of the integrated risk addends arising from testing and estimation allows moving smoothly between these two targets. Furthermore, we explore different possible elicitations of the test error costs, leading to test decisions based either on posterior probabilities, or solely on Bayes factors. Sensitivity analyses are performed following the convention which makes a distinction between the prior of the data-generating process, and the analysis prior adopted to fit the data. Simulation in the case of normal and binomial outcomes and an application to a one-arm proof-of-concept trial, exemplify how such analysis can be conducted to explore sensitivity of the integrated risk, the operating characteristics, and the optimal sample size, to prior-data conflict. Robust analysis prior specifications, which gradually discount potentially conflicting prior information, are also included for comparison. Guidance with respect to cost elicitation, particularly in the context of a Phase II proof-of-concept trial, is provided.

## 1. Introduction

Bayesian clinical trial designs are often evaluated in terms of frequentist (i.e., conditional on a given parameter value) operating characteristics such as type I error rate, power, and mean squared error (MSE). The work of Viele *and others* (2014) provides an overview of the effects of different borrowing mechanisms on clinical trial frequentist operating characteristics. In general, improvements in terms of frequentist type I error rate, power, and MSE can be achieved if prior (historical) information is indeed consistent with the true parameter value generating the current data, at the cost of type I error rate and MSE inflation, and power loss, otherwise. Indeed, if strict control of frequentist type I error rate is required and a uniformly most powerful test is available, the maximum attainable power ultimately depends only on the final sample size, irrespectively of whether a static or dynamic borrowing mechanism is adopted, i.e., it does not depend on whether the prior is fixed or adapts to the observed degree of conflict between historical and current information (see Kopp-Schneider *and others*, 2020). In practice, however, a carefully elicited prior should contain trustworthy information, and its incorporation is therefore likely to improve the trial’s test decision and effect estimate. It is therefore of interest to investigate the rationale and consequences of different approaches to relaxing strict control of frequentist operating characteristics in a quantitative, rational, and unified framework. Moreover, as sampling generally comes at a non-negligible monetary and/or ethical cost, such a framework should allow taking sampling costs into account when eliciting the trial’s sample size.

The task just described is essentially a Bayesian decision-theoretic problem. In this context, we define a loss function to explicitly account for costs in terms of all operating characteristics, including the estimation error, and in terms of sample size. Estimation error can be of particular relevance in, e.g., a Phase II proof-of-concept trial. Indeed, such a trial aims at assessing both significance and relevance of a treatment, and can more likely result in indeterminate outcomes (i.e., when only significance or relevance is declared, see e.g., Fisch *and others*, 2014) also under a large true effect if a large uncertainty is associated with the posterior estimate. In addition, as the posterior estimate of a Phase II trial is often used to inform the sample size calculations of the subsequent Phase III trial, a large estimation error can induce a high risk of an under- or over-powered Phase III trial (Wang *and others*, 2006).

We show how the decision-theoretic framework can unify different approaches to sample size selection, ultimately connected to cost elicitation. Moreover, following Sahu and Smith (2006), we illustrate how sensitivity analyses can be performed through the elicitation of two priors: a sampling prior, which is used to reproduce different potential data-generating mechanisms, and an analysis prior, which is used to fit the data and perform the trial. The sampling and the analysis priors are also referred to in the literature as the design and the fitting prior, respectively. In addition to the performance of sensitivity analysis, their distinction has been adopted to reflect a different view on the use of prior information. For example, a pharmaceutical company may be willing to incorporate available prior information to estimate the probability of success of a trial (also known as assurance or average power), while regulatory agencies may require the analysis to be performed under a pessimistic prior or the frequentist paradigm (O’Hagan and Stevens, 2001; Crisp *and others*, 2018). Indeed, the sampling prior allows formally taking into account uncertainty about the parameter of the data-generating process when computing the operating characteristics of the design (O’Hagan and Stevens, 2001). On the other hand, frequentist operating characteristics can still be retrieved simply by assuming a point-mass sampling prior.

In contrast to the commonly adopted frequentist approach to type I error rate control, however, here we place particular focus on the idea of controlling a cost-weighted sum of both error rates (see e.g., Grieve, 2015; Pericchi and Pereira, 2016). Such choice has decision-theoretic foundations, and has been shown to unify Bayesian and frequentist approaches to hypothesis testing by effectively solving Lindley’s paradox (i.e., the fact that different test decisions are provided by frequentist and Bayesian approaches as the sample size increases) (Pericchi and Pereira, 2016); moreover, it induces a test decision strictly related to the value of the Bayes factor (BF), which has been advocated as a sensible alternative to $p$-values as a measure of statistical evidence (Bayarri *and others*, 2016).

Section 2 introduces the decision-theoretic framework adopted in this work, it outlines the rationale for the control of a weighted sum of error rates, and provides further details on the choice and consequences of the sampling-analysis prior dichotomy. Section 3 describes the proposed approach by introducing the target loss function and provides guidance on cost elicitation. The simulations of Section 4 exemplify its behavior in terms of operating characteristics and elicited sample sizes for different analysis prior specifications, including robust prior specifications. Section 5 provides a sample application to a proof-of-concept trial (Roychoudhury *and others*, 2018). In the final section, we provide a summary of this work, an outlook on possible developments, and a further endorsement of the decision-theoretic approach in the context of the recent debate on $p$-values and significance testing.

## 2. The decision-theoretic framework

### 2.1. Hypothesis testing

Assume that a clinical trial is performed to test the null hypothesis $H_0$ that a given effect $\theta \leq \theta_0$ versus the alternative hypothesis $H_1$ that $\theta> \theta_0$. Let $\mathbf{y}$ denote the observed data having probability density function $f(\mathbf{y}|n,\theta)$ indexed by $\theta$ and sample size $n$, and let $\pi(\theta)$ be the prior distribution for $\theta$. Let also $m(\mathbf{y})$ denote the marginal data distribution, i.e. $m(\mathbf{y})=\int_\Theta f(\mathbf{y}|n,\theta) \pi(\theta) {\rm d}\theta$. The decision $d_{c_0,c_1}$ consists in either rejecting ($d_{c_0,c_1}=1$) or keeping ($d_{c_0,c_1}=0$) the null hypothesis. A $c_0$–$c_1$ loss function can be defined as (see e.g., Robert, 2007)

(2.1) \begin{equation*} L(\theta, d_{c_0,c_1}(\mathbf{y}))= \begin{cases} 0 & \text{if} \ d_{c_0,c_1}(\mathbf{y})=I_{\{\theta > \theta_0\}}\\ c_0 & \text{if} \ I_{\{\theta > \theta_0\}} \ \& \ d_{c_0,c_1}(\mathbf{y})=0\\ c_1 & \text{if} \ I_{\{\theta \leq \theta_0\}} \ \& \ d_{c_0,c_1}(\mathbf{y})=1, \end{cases} \label{eq:utilityDef} \end{equation*}

where $I_{}$ denotes the indicator function, and $c_0$ and $c_1$ are the losses assigned to keeping $H_0$ when it is false and rejecting $H_0$ when it is true, respectively. Taking into account uncertainty about $\theta$, the optimal test decision after an outcome $\mathbf{y}$ has been observed is obtained by minimization of the posterior expected loss $\rho(\pi, d_{c_0,c_1}(\mathbf{y})| \mathbf{y})=E^{\pi} [L(\theta, d_{c_0,c_1}(\mathbf{y}))|\mathbf{y}]$, i.e.,

(2.2) \begin{align*} \label{eq:exloss} \rho(\pi, d_{c_0,c_1}(\mathbf{y})| \mathbf{y})= & c_1 P^{\pi}(\theta \leq \theta_0|\mathbf{y}) I_{\{d_{c_0,c_1}(\mathbf{y})=1\}} + c_0 \{1-P^{\pi}(\theta \leq \theta_0|\mathbf{y})\} I_{\{d_{c_0,c_1}(\mathbf{y})=0\}}, \end{align*}

where $P^{\pi}(\theta \leq \theta_0|\mathbf{y})$ is the posterior probability of the null hypothesis under data $\mathbf{y}$ and prior $\pi$. $\rho(\pi, d_{c_0,c_1}(\mathbf{y})| \mathbf{y})$ is minimized by choosing $d_{c_0,c_1}^{\pi}(\mathbf{y})= I_{\{P^{\pi}(\theta \leq \theta_0|\mathbf{y})<\gamma^{\pi} \}}$, where $\gamma^{\pi}= c_0/(c_0+c_1)$ (note that only the ratio between $c_0$ and $c_1$ is relevant). In contrast, uncertainty about $\mathbf{y}$ can be taken into account by averaging the loss function (2.1) across data realizations for a fixed $\theta$, leading to the frequentist risk measure $ R(\theta, d_{c_0,c_1}(\mathbf{y}))=E^{f} [L (\theta, d_{c_0,c_1}(\mathbf{y}))]$,

(2.3) \begin{align*} \label{eq:riskFn}R(\theta, d_{c_0,c_1}(\mathbf{y}))=& c_1 I_{\{\theta \leq \theta_0\}} P^f(d_{c_0,c_1}(\mathbf{y})=1|\theta) + c_0 I_{\{\theta >\theta_0\}} \{1-P^f(d_{c_0,c_1}(\mathbf{y})=1|\theta)\}, \end{align*}

where $P^f(d_{c_0,c_1}(\mathbf{y})=1|\theta)$ is the probability of rejecting $H_0$ under the data density $f(\mathbf{y}|n, \theta)$. Note that, for $c_0=c_1=1$, the risk function (2.3) represents the conditional type I error rate $\alpha(\theta)$ for $\theta \leq \theta_0$, or type II error rate $\beta(\theta)=1-\alpha(\theta)$, for $\theta>\theta_0$. Uncertainty about both the data outcome and the parameter value can be accounted for by integration of the frequentist risk (2.3) over $\pi(\theta)$ or, equivalently, of the posterior expected loss (2.2) over the marginal data distribution $m(\mathbf{y})$. This leads to the integrated risk measure

(2.4) \begin{align*} r(\pi, d_{c_0,c_1}(\mathbf{y}))=&\int_{-\infty}^{\theta_0} c_1 \alpha(\theta) \pi(\theta) {\rm d}\theta + \int_{\theta_0}^{\infty} c_0 \beta(\theta) \pi(\theta){\rm d}\theta. \label{eq:intrisk} \end{align*}

Therefore, the decision $d_{c_0,c_1}^{\pi}(\mathbf{y})$ which minimizes the posterior expected loss for each $\mathbf{y} \in Y$, also minimizes the integrated risk (see, e.g., Robert, 2007). Note that if $\pi(\theta)$ is chosen as a two-point mass prior, with one point belonging to the support of the null, and one point belonging to the support of the alternative hypothesis, the integrated risk (2.4) is equivalent to the frequentist risk (2.3) with “re-weighted” $c_0$ and $c_1$. The integrated risk (2.4) can also be seen as the sum of the average type I error rate $\alpha=\int_{-\infty}^{\theta_0} \alpha(\theta) \{P^{\pi}(\theta \leq \theta_0)\}^{-1} \pi(\theta) {\rm d}\theta$ and type II error rate $\beta=\int_{\theta_0}^{\infty} \beta(\theta) \{P^{\pi}(\theta > \theta_0)\}^{-1} \pi(\theta) {\rm d}\theta$, by taking $c_0 = \{P^{\pi}(\theta > \theta_0)\}^{-1}$ and $c_1= \{P^{\pi}(\theta \leq \theta_0)\}^{-1}$.

#### Minimizing the sum of type I and type II error rate

The optimal decision $d_{c_0,c_1}^{\pi}$ is intrinsically connected to the value of the BF. Indeed, $H_0$ is kept if $P^{\pi}(\theta \leq \theta_0|\mathbf{y}) \geq \gamma^{\pi}$, i.e., if (see e.g., Parmigiani and Inoue, 2009; Pericchi and Pereira, 2016)

(2.5) \begin{equation*} \frac{c_0}{c_1} \leq \frac{P^{\pi}(\theta \leq \theta_0)}{P^{\pi}(\theta > \theta_0)} \frac{\int_{-\infty}^{\theta_0} f(\mathbf{y}| \theta) \pi(\theta| \theta \in (-\infty,\theta_0]) {\rm d} \theta}{\int_{\theta_0}^{\infty} f(\mathbf{y}| \theta) \pi(\theta| \theta \in (\theta_0,\infty)) {\rm d} \theta} \label{eq:decisionBF} \end{equation*}

The first factor on the right-hand side of Equation (2.5) is given by the prior odds of the null versus the alternative hypothesis, while the second factor is the BF of the null versus the alternative hypothesis. By exploiting the duality between prior probabilities and test error costs (Robert, 2007), if we assume $c_0=c_0' \{P^{\pi}(\theta > \theta_0)\}^{-1}$ and $c_1=c_1' \{P^{\pi}(\theta \leq \theta_0)\}^{-1}$, $H_0$ is then kept if (Pericchi and Pereira, 2016)

(2.6) \begin{equation*} \frac{c_0'}{c_1'} \leq \frac{\int_{-\infty}^{\theta_0} f(\mathbf{y}| \theta) \pi(\theta| \theta \in (-\infty,\theta_0]) {\rm d} \theta}{\int_{\theta_0}^{\infty} f(\mathbf{y}| \theta) \pi(\theta| \theta \in (\theta_0,\infty)) {\rm d} \theta} = BF(\mathbf{y}), \label{eq:decisionBF2} \end{equation*}

i.e., if the ratio of the costs is lower than the BF. The advantage of the latter cost elicitation is that the integrated risk can be seen as the (weighted) sum of the average type I and type II error rates, which should aid interpretability and cost elicitation. Moreover, the test decision no longer depends on the prior odds. In this sense, it can be thought to be induced only by the available data, although the influence of the prior shape distinguishes it from a purely frequentist concept unless point-mass priors are adopted (Berger and Sellke, 1987).

Performing a test decision based only on the principle of minimizing the sum of test error rates, thus without enforcement of (either conditional or average) type I error rate control, is the natural and optimal choice in a decision-theoretic set-up, and has been advocated in various works (see, e.g., Grieve, 2015; Pericchi and Pereira, 2016; Isakov *and others*, 2019, and references therein). As expressed very clearly in Pericchi and Pereira (2016) and references therein, an error rates-weighted approach defines the rejection region by fixing the required amount of evidence in favor of the alternative hypothesis relative to the null, rather than by fixing the type I error rate, and this has desirable consequences in terms of asymptotic behavior and independence of the result from the mechanism by which data were collected. Crucially, the latter also implies that no adjustment is required in sequential testing procedures (Grieve, 2015; Bayarri *and others*, 2016). Additionally, Pericchi and Pereira (2016) show that Lindley’s paradox is not a discrepancy between frequentist and Bayesian test decisions, but rather a discrepancy between decisions based on fixing type I error rate, and decisions which allow a decrease in both test error rates, according to their relative costs, as the sample size increases. It is also of interest to note that (see Bayarri *and others*, 2016; Pericchi and Pereira, 2016) $\int_Y BF(\mathbf{y}) f(\mathbf{y}| \theta \in (\theta_0,\infty), R) {\rm d}y = \alpha/(1-\beta)$, where $R$ denotes the rejection region, i.e., the set of $\mathbf{y}$ outcomes such that $d_{c_0,c_1}^{\pi}(\mathbf{y})=1$, and $\alpha$ and $\beta$ are the average type I and type II error rate, respectively. This is directly related to Equation (2.6), and in particular to the fact that the decision based on the BF is the minimizer of the sum of average test error rates (Pericchi and Pereira, 2016).

### 2.2. Sampling and analysis prior

As outlined in the Section 1, here we assume that two priors are elicited, a sampling prior $\pi_s$, which is assumed to generate the data, and an analysis prior $\pi_a$, which is used to fit the data and which induces the actual trial decisions (see, e.g., O’Hagan and Stevens, 2001; De Santis, 2006; Sahu and Smith, 2006; Psioda and Ibrahim, 2018). Assuming the integrated risk (2.4) to be computed with respect to the sampling prior $\pi_s$, it would be minimized by a choice taken with respect to the sampling prior itself, i.e., $d^{\pi_s}_{c_0,c_1}(\mathbf{y})$. In practice, however, the true sampling prior is unknown and thus decisions have to be undertaken based on optimality with respect to the analysis prior. Sensitivity analyses can be performed to explore the integrated risks induced by different data-generating mechanisms. Following Sahu and Smith (2006), the integrated risk is then computed as $r(\pi_s, d^{\pi_a}_{c_0,c_1}(\mathbf{y}))=E^{\pi_s} [R(\theta, d^{\pi_a}_{c_0,c_1}(\mathbf{y}))]$.

In the formalization of Berger (1990), our and Sahu and Smith’s (2006) approach performs sensitivity analyses with respect to the integrated risk as functional of interest. Note that we obtain the integrated risk by averaging the frequentist risk over the sampling prior distribution (rather than averaging the posterior expected loss induced by the analysis prior over the marginal data distribution). When $\pi_s \neq \pi_a$, the two approaches would not lead to the same result. Our choice has the desirable feature of providing a straightforward connection with the frequentist approach simply by replacing the sampling prior with a point-mass prior. Furthermore, in a context where no dichotomy between sampling and analysis prior is assumed, Berger (1985) notes that this approach involves *“uncertainty about $\pi$ only at the stage of averaging $R(\theta, d).$”*

If sensitivity analyses are not of primary interest, a single sampling prior can be elicited (see, e.g., Psioda and Ibrahim, 2018). In the *default* sampling prior approach of Psioda and Ibrahim (2018), the sampling priors under the null and alternative hypothesis arise by truncation of the prior elicited from the historical information at $\theta_0$, and the subsequent normalization. Average type I error rate and power are then computed with respect to the elicited sampling priors, and their control is achieved by adopting a fixed threshold for rejection, equal to the desired average type I error rate, and adapting the analysis prior informativeness and the sample size until the desired characteristics are obtained.

Here, we follow a related approach which, however, includes the costs of average rates of type I and type II errors in the integrated risk function and targets the weighted sum of such average test error rates. Moreover, we incorporate estimation error and sampling costs as criteria to derive an optimal sample size. Finally, the distinction between $\pi_s$ and $\pi_a$ is exploited to perform sensitivity analyses of the optimal sample size and operating characteristics with respect to different data-generating processes conveyed by the sampling prior $\pi_s$.

## 3. Extended integrated risk definition and optimisation

### 3.1. Extended integrated risk definition

Early phase clinical trials, e.g., proof of concept studies, often focus not only on detecting efficacy, i.e., whether the null hypothesis can be rejected, but also on determining relevance, i.e., whether the observed effect is large enough to motivate proceeding to a Phase III study. Here, we follow Fisch *and others* (2014) and denote a trial outcome “relevant” if $P^{\pi_a}(\theta \leq \theta_R|\mathbf{y}) < 0.5$, where $\theta_R$ is the relevance threshold, and “indeterminate” if either relevance or significance is observed. When $\theta > \theta_R$, we expect the probability of an indeterminate outcome to correlate with estimation error as the sample size increases (we will show in Section 4 that such probability may, however, be non-monotone). Estimation error might be considered even more harmful if, as is often the case, the effect detected in the Phase II trial is exploited to perform sample size calculations for the subsequent Phase III trial, as a large error can induce a significantly higher risk of Phase III trial under- or over-powering (see Wang *and others*, 2006, for a discussion and a related approach addressing this point). These observations motivate the introduction of estimation error in the loss function (2.1). Thereby, we add to each subcase the commonly adopted quadratic loss function with decision $d_q$, $L(\theta, d_{q}(\mathbf{y}))= \{\theta-d_{q}(\mathbf{y})\}^2$ (see, e.g., Parmigiani and Inoue, 2009). Moreover, as both the test error rates and the estimation error would decrease for increasing sample size, a desirable principled approach for the choice of the sample size needs to explicitly incorporate the cost of each additional observation into the loss function (see, e.g., Lindley, 1997). The cost of sampling can be assumed to be, e.g., additive and linear in the number of observations (Lindley, 1997). The integrated risk (2.4) becomes

(3.7) \begin{align*} \label{eq:intrisk3} r(n)=& \int_{-\infty}^{\theta_0} c_1 \alpha(\theta) \pi_s(\theta) {\rm d}\theta + \int_{\theta_0}^{\infty} c_0 \beta(\theta) \pi_s(\theta) {\rm d}\theta + c_q \int_{\Theta} MSE(\theta) \pi_s(\theta) {\rm d}\theta +c_n n, \end{align*}

where ${\rm MSE}(\theta)=E^f[\theta-d^{\pi_a}_{q}(\mathbf{y})]^2$, $c_q$ is a constant introduced to reflect the relative importance of estimation versus test error, and $c_n$ is the cost per sample. Note that we have dropped the dependence of $r$ on the sampling prior $\pi_s$ and the vector of decisions with respect to the analysis prior $\mathbf{d^{\pi_a}}(\mathbf{y})=\{d^{\pi_a}_{c_0,c_1}(\mathbf{y}), d^{\pi_a}_{q}(\mathbf{y})\}$ for ease of notation. When $\pi_a=\pi_s$, the risk is minimized by the optimal choices $d^{\pi_a}_{c_0,c_1}(\mathbf{y})$ and $d^{\pi_a}_{q}(\mathbf{y})=E^{\pi_a}[\theta|\mathbf{y}]$, while the optimal sample size needs to be identified numerically.

### 3.2. Cost elicitation

Cost elicitation can proceed as follows. We can first select $c_1=c_1' \{P^{\pi_s}(\theta \leq \theta_0)\}^{-1}$ and $c_0=c_0' \{P^{\pi_s}(\theta > \theta_0)\}^{-1}$ according to the relative importance assigned to each average error rate, keeping in mind that the BF should be larger than $c_0'/c_1'$ to keep $H_0$ (see, e.g., Kass and Raftery, 1995; Pericchi and Pereira, 2016, for guidance on BF thresholding); to facilitate interpretation, we can additionally require $c'_1+c'_0=1$. Note that, if we aim at minimizing the weighted sum of average test error rates, under the sampling prior we have that $c_0=c_0' \{P^{\pi_s}(\theta > \theta_0)\}^{-1}$ and $c_1=c_1' \{P^{\pi_s}(\theta \leq \theta_0)\}^{-1}$. However, as the sampling prior is generally not available, decisions must be undertaken with respect to the analysis prior, leading to a threshold for rejection $\gamma^{\pi_a}=\frac{c_0' P^{\pi_a}(\theta \leq \theta_0)}{c_0' P^{\pi_a}(\theta \leq \theta_0)+c_1' (1-P^{\pi_a}(\theta \leq \theta_0))}$. As $\pi_a$ approaches $\pi_s$, $\gamma^{\pi_a}$ also approaches the optimal decision threshold $\gamma^{\pi_s}$, and the integrated risk is minimized. However, for the purpose of evaluating the integrated risk (3.7), $c_0$ and $c_1$ are computed with respect to the sampling prior, and only the decision is affected by $\pi_a$.

To elicit $c_q$ and $c_n$, we take advantage of the connection between the “goal sampling” and the integrated risk minimization approach highlighted by Lindley (1997). In particular, for a specific sample size choice induced by the goal sampling approach, i.e., the minimum sample size required to achieve specific testing or estimation targets, a cost per observation can be inferred through a local approximation of the integrated risk. This can be achieved by first noting that both operating characteristics, i.e., weighted sum of average test error rates and average MSE, are a function of the sample size $n$. Let the integrated risk for, e.g., the weighted sum of average test error rates (SATE) be denoted by $r^{\rm SATE}(n)={\rm SATE}(n)+c^{\rm SATE}_n n$. In the usual risk optimization procedure, $c^{\rm SATE}_n$ is fixed, while the sample size and, therefore, ${\rm SATE}(n)$ are varied so that the integrated risk is minimized. However, if a target SATE is specified and a given sample size $n^{\rm SATE}$ is elicited so that such SATE target is achieved, the cost per observation corresponding to this combination can be inferred. In particular $r^{\rm SATE}(n)$ is minimized if ${\rm d} r^{\rm SATE}(n)/ {\rm d}n= 0$, i.e., if $c^{\rm SATE}_n= - \frac{{\rm d SATE}(n)}{{{\rm d}}n}\rvert_{n^{\rm SATE}}$. The same procedure can be applied to infer a cost for the average MSE (AMSE), $c^{\rm AMSE}_n$. An example is provided in Section S1 and Figure S1 of the supplementary material available at *Biostatistics* online. This procedure also highlights why goal sampling approaches cannot be considered “cost free,” and are rather based on an implicit elicitation of the costs (Lindley, 1997).

In order to obtain $c_q$ and $c_n$ in Equation (3.7), note that the minimum of the integrated risk is invariant to multiplication by a constant; therefore, if we divide $r^{\rm SATE}(n)$ and $r^{\rm AMSE}(n)$ by $c^{\rm SATE}_n$ and $c^{\rm AMSE}_n$, respectively, the combined integrated risk (3.7) can be written as

(3.8) \begin{align*} \label{eq:utilityInt2} r(n)=& \frac{w}{c^{\rm SATE}_n} \left\{ \int_{-\infty}^{\theta_0} c_1 \alpha(\theta) \pi_s(\theta) {\rm d}\theta + \int_{\theta_0}^{\infty} c_0 \beta(\theta) \pi_s(\theta) {\rm d}\theta \right\} + \frac{(1-w)}{c^{\rm AMSE}_n} \int_{\Theta} MSE(\theta) \pi_s(\theta) {\rm d}\theta + n, \end{align*}

which is equivalent to assuming $c_q=\{(1-w) c^{\rm SATE}_n\}/\{w c^{\rm AMSE}_n\}$ and $c_n = c^{\rm SATE}_n/w$. The weight $w \in [0,1]$ has the role of defining the importance of the testing versus the estimation target. We propose taking $w=n^{\rm SATE}/(n^{\rm SATE}+n^{\rm AMSE})$, under the implicit assumption that the importance of each characteristic is reflected in the number of patients one would be willing to pay for the corresponding target. Note that the sample size minimizing the integrated risk (3.8) will lie between $n^{\rm SATE}$ and $n^{\rm AMSE}$: If the targets specified are to be strictly satisfied, then one should adopt a pure goal sampling approach and select $n$ as the maximum between $n^{\rm SATE}$ and $n^{\rm AMSE}$.

The derivatives of ${\rm SATE}(n)$ and ${\rm AMSE}(n)$ may not be analytically available. A numerical approximation is however easily available once values for both operating characteristics are computed in a neighborhood of their respective target sample sizes. We therefore suggest to compute ${\rm SATE}(n)$ and ${\rm AMSE}(n)$ for increasing sample sizes under a chosen sampling prior and analysis prior; these can be reasonably taken to be, e.g., the available informative prior and the intended analysis prior, respectively. At this stage, the average type I and type II error rates can also be stored to simplify elicitation of a target weighted sum of average test error rates. To simplify elicitation of a target AMSE, the average probability of an indeterminate outcome, the average “extreme” power loss and the average “extreme” sample size gain when $\theta > \theta_R$, can also be computed. Extreme power loss and sample size gain could be expressed, for example, in terms of the $(1-\zeta)\%$ quantiles of the respective conditional distributions induced by the data outcomes. We define the power loss and sample size gain as the difference between a target Phase III power (0.9, for example) and corresponding sample size, and the “realized” power and sample size, respectively. That is, they are defined based on standard frequentist sample size calculations if the posterior mean of the current trial is taken as the “true” effect. When the trial result is not significant and/or not relevant, the assumption is that the realized power and sample size are equal to 0, as the Phase III trial is not taking place. The conditional probability of an indeterminate outcome, and the $(1-\zeta)\%$ quantiles of power loss and sample size gain, are then averaged with respect to the sampling prior distribution truncated from below at $\theta_R$, i.e., we only concentrate on losses for $\theta > \theta_R$. Once the costs have been calculated, the integrated risk is readily available for a grid of sample sizes, and the final optimal sample size can be numerically identified as the one minimizing the overall risk. Alternatively, if available, actual monetary values can be assigned to all costs.

## 4. Evaluation: designing a trial and checking sensitivity to prior-data conflict

To illustrate the concepts and the proposed approaches, we first focus on a normal outcome $\mathbf{y}$, $y_i \sim N(\theta, \sigma^2=1)$, $i=1,\dots,n$. Interest is placed on the mean parameter $\theta$, which is assumed to follow a $N(\mu_s,\sigma^2_s)$ sampling prior. We test the set of hypotheses $H_0: \theta \leq 0$ versus $H_1: \theta > 0$, and assume a relevance threshold $\theta_R=0.15$. The following analysis priors are considered: (i) A vague prior $\pi_{\rm vague}$, which follows a $N(0,10^2)$ distribution; (ii) an informative prior, which follows a $N(0.25,1/50)$ distribution; (iii) a robust mixture prior (see, e.g., Berger and Berliner, 1986; Schmidli *and others*, 2014), which is a mixture of the informative prior and a vague $N(0.25,10^2)$ prior distribution, with mixture weight equal to 0.5; and (iv) an empirical Bayes (EB) power prior $\pi_{\rm pow}$. The EB power prior can be interpreted as a posterior arising from a historical study which collected $n_0$ observations, of which $a_0 n_0$ are accounted for in the analysis, where the parameter $a_0 \in [0,1]$ is selected by maximization of the marginal likelihood $m(\mathbf{y})=\int f(\mathbf{y} |n, \theta) \pi(\theta|a_0,\mathbf{y_0}, n_0) {\rm d}\theta$, where $\pi(\theta|a_0,\mathbf{y_0}, n_0)$ denotes the power prior with parameter $a_0$, and $\mathbf{y_0}$ and $n_0$ are the historical outcome and sample size, respectively (Gravestock and Held, 2017). Here, the historical outcome is summarized by the mean $\bar{y}_0=0.25$, and the historical sample size is $n_0=50$. When $a_0=1$, the EB power prior coincides with the informative prior. Note that the EB commensurate prior (Hobbs *and others*, 2012) for normal outcomes coincides with the EB power prior (see Appendix C.1 of Wiesenfarth and Calderazzo, 2020), and therefore all results for the EB power prior directly apply also to the EB commensurate prior in this set-up.

**Fig. 1.**
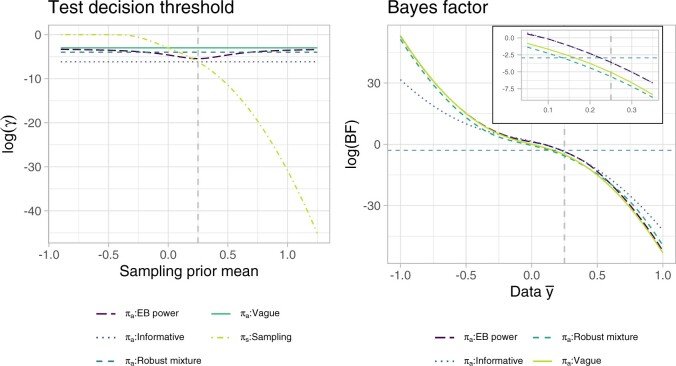
Decision threshold $\gamma^\pi$ for different prior specifications (left), and BF (right), on the logarithmic scale. The sample size is assumed equal to 100 in both plots. For the test decision threshold plot, the sampling prior variance is fixed at $\sigma^2_s=1/50$. The dashed vertical line corresponds to the point at which the sampling prior and the informative prior coincide. In the BF plot, the dashed horizontal line corresponds to $\log(c'_0/c'_1)$, $c_0'=0.05,$ and $c_1'=0.95$, i.e., the log- (BF) threshold for rejection, see Equation (2.6).

We assume that type I error is 19 times more costly than type II error, thus we take $c_0'=0.05$ and $c_1'=0.95$ . To gain further insight into the analysis prior decision thresholds and test decisions, Figure 1 provides an illustration of each decision threshold $\gamma^{\pi_{\rm prior}}$ for varying sampling prior mean $\mu_s$ ($\sigma^2_s=1/50$), and of the BF for varying data mean outcomes $\bar{y}$, assuming $n=100$. The decision thresholds of all analysis priors tend to differ more from the sampling prior optimal threshold for negative values of $\mu_s$ (values are plotted on the logarithmic scale for ease of representation), which is indeed the region where the historical information differs most from the sampling prior; the EB power prior threshold depends also on the data $\bar{y}$ and varies, as expected, between the vague and the informative prior ones, depending on the degree of conflict. Note that for the vague prior $P^{\pi_{\rm vague}}(\theta \leq \theta_0) \approx 0.5$, thus $\gamma^{\pi_{\rm vague}}\approx c'_0/(c'_0+c'_1)$. Moreover, as $\sigma_{\rm vague} \rightarrow \infty$, $d^{\pi_{\rm vague}}_{c_0,c_1}$ approaches the usual frequentist test decision at level 5%. As for the BF, it is of interest to note that, in the region close to the threshold $c'_0/c'_1$, the EB power prior BF behaves very similarly to the informative prior one, while the mixture prior BF is closer to the vague prior BF. This has to be partly attributed to the shape of the corresponding null and alternative hypothesis truncated densities. Sensitivity of the BF to the location of historical information and weight and vague component of the robust mixture prior is investigated in Section S2 and Figure S2 of the supplementary material available at *Biostatistics* online.

**Fig. 2.**
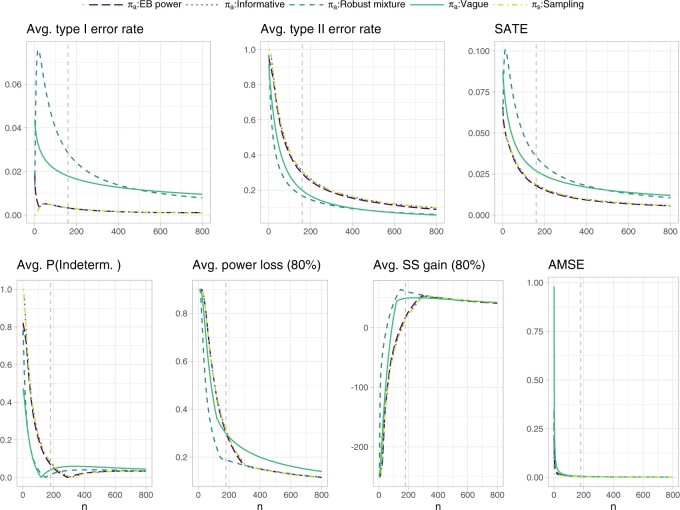
Operating characteristics related to testing and estimation for the normal outcome simulation example. The extreme power loss and extreme sample size gain in a subsequent Phase III trial, are defined as the 80% quantile of the respective distributions induced by the data outcomes. The probability of an indeterminate outcome, the extreme power loss and extreme sample size gain are averaged with respect to the sampling prior $N(0.25,1/50)$ truncated from below at $\theta_R=0.15$. The remaining operating characteristics are averaged with respect to the whole sampling prior distribution. The dashed vertical lines represent the sample sizes identified for cost elicitation with respect to the vague analysis prior, and, independently, for testing (upper panels, $n^{\rm SATE}=160$) and estimation (lower panels, $n^{\rm AMSE}=180$). Such sample sizes allow to maintain an average type I and type II error rate below 0.05 and 0.2, respectively, as well as an average probability of an indeterminate outcome, average extreme power loss and average extreme sample size gain below 0.1, 0.3, and approximately 50, respectively. Note that the informative prior results exactly overlap with the sampling prior ones.

The costs $c^{\rm SATE}_n$ and $c^{\rm AMSE}_n$ are derived as described in Section S1 of the supplementary material available at *Biostatistics* online, where $n^{\rm SATE}=160$ and $n^{\rm AMSE}=180$ are elicited through inspection of Figure 2 as follows. The sampling prior is assumed to coincide with the informative prior specification. Further we assume, e.g., that the vague prior was selected as analysis prior. Assume that, to reach an average type II error rate below 0.2 under the vague analysis prior specification, one is willing to “pay” the required $n^{\rm SATE}=160$ samples. The average type I error rate is in this case always below 0.05, so we assume it does not contribute to the sample size requirement. This sample size corresponds to a weighted sum of average test error rates of 0.027, therefore we have $c^{\rm SATE}_n= 8.511 \cdot 10^{-5}$. Analogously, suppose the required $n^{\rm AMSE}=180$ samples are deemed acceptable to reach an average probability of an indeterminate outcome under $\theta > \theta_R$ below 0.1 and an average extreme power loss (we take the $1-\zeta=0.8$ quantile of the data induced distribution) and average extreme sample size gain (again for $1-\zeta=0.8$) below 0.3 and approximately 50, respectively. This would correspond to an AMSE equal or lower than 0.006, and cost $c^{\rm AMSE}_n= 3.098 \cdot 10^{-5}$. Finally, the weight $w=n^{\rm SATE}/(n^{\rm SATE}+n^{\rm AMSE})$ results in 0.471. In principle, a different choice of costs can be associated with each analysis prior. This would however result in integrated risks which are no longer directly comparable across different analysis priors. Therefore, to simplify our exposition, we retain the costs elicited under the vague analysis prior for all comparisons. Note, also, that the probability of an indeterminate outcome is not monotone for the vague and robust mixture analysis priors: this is due to the fact that, for low sample sizes, relevance can be achieved without significance; as the sample size increases, relevance tends to be achieved whenever also significance is observed; the probability of an indeterminate outcome slightly rises again as the sample size becomes large enough to allow significance without relevance, to then decrease again for larger sample sizes as a relevant outcome becomes more likely. Such potentially non-monotonic behavior makes the probability of an indeterminate outcome not ideal for the purposes of defining our costs, but we still regard it as of general interest for the design of the trial, and thus we believe it should be additionally monitored at the cost elicitation stage.

**Fig. 3.**
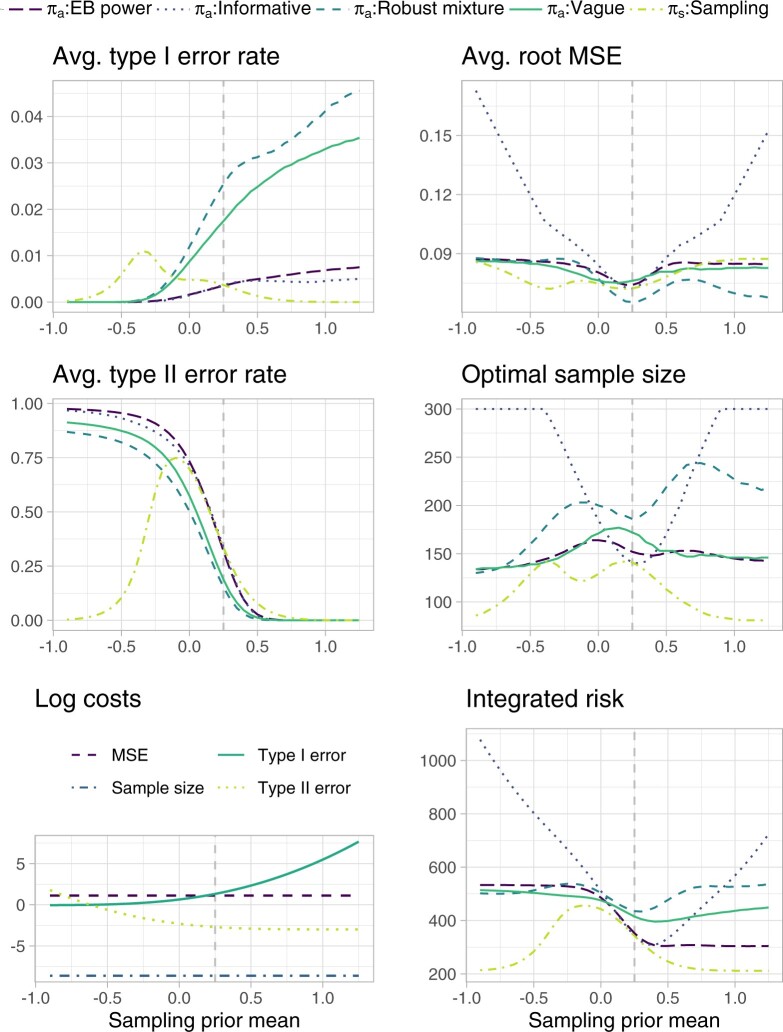
Integrated risk, costs, and operating characteristics for different analysis prior specifications and for varying sampling prior means $\mu_s$. The sampling prior variance is fixed at $\sigma^2_s=1/50$, and the sample size is optimized to minimize the integrated risk. Costs are elicited as follows: $c_0=c_0' (1-P^{\pi}[\theta \leq \theta_0])^{-1}$, $c_1=c_1' (P^{\pi}[\theta \leq \theta_0])^{-1}$, $c_q=\{(1-w) c^{\rm SATE}_n\}/\{w c^{\rm AMSE}_n\},$ and $c_n = c^{\rm SATE}_n/w$ (see Equation (3.8)), where $c_0'=0.05$, $c_1'=0.95$, $c^{\rm SATE}_n= 8.511 \cdot 10^{-5}$, $c^{\rm AMSE}_n= 3.098 \cdot 10^{-5}$, and $w=0.471$. Note that test error log costs refer to the sampling prior specification. The dashed vertical line corresponds to the point at which the sampling prior and the historical information coincide. The maximum sample size is truncated at n=300.

Once costs have been selected, the sample size is selected through minimization of the integrated risk, and sensitivity analyses can be carried out by varying the sampling prior specification. Figure 3 shows how each of the considered operating characteristics, the costs, and the integrated risk behave when $\sigma^2_s=1/50$, while varying $\mu_s$. As a consequence of the BF behavior illustrated in Figure 1, the EB power and the informative prior tend to attain very close average test error rates; the same can be observed to a lesser extent for the vague and robust mixture prior. The AMSE tends to be lower when the historical information and the sampling prior agree, for all priors incorporating historical information. The vague prior shows a decreased AMSE, coupled with an increased sample size, in a nearby region close to 0. This is induced by the fact that in this region it is harder to discriminate between the two hypotheses. Focusing on the costs, when $\mu_s << 0$, the cost of a type II error is higher than the cost of a type I error, as the sampling prior is placing a low probability on the alternative hypothesis. We stress again that this slightly counter-intuitive cost definition is effectively aimed at basing the test choice only on the comparison between $c'_0/c'_1$ and the BF, as shown in Equation (2.6). As $\mu_s$ increases, the cost of a type II error decreases, while the cost of a type I error increases. The imbalance in the maximum cost of each error is due to the fact that we have specified $c'_1$ to be much larger than $c'_0$. The overall integrated risk shows non-robustness of the informative prior, reflecting the high sample size requirement and a steep increase in AMSE. The EB power prior reaches a remarkably small integrated risk when $\mu_s >>0$, due to the fact that it achieves average test error rates close to the informative prior, while constraining losses in term of AMSE. On the negative support of $\mu_s$ this behavior is reversed, i.e., the EB power prior shows the highest integrated risk after the informative prior one, although less strikingly so, due to the imbalance in the test error costs. In terms of overall integrated risk, the robust mixture prior appears less robust than the EB power prior; this is also reflected in the sample size requirement and is mostly induced by the behavior of the average test error rates. Note that the result is influenced by the choice of the mixture weight, the vague component, and the location of historical information. We refer again to Section S2 and Figure S2 of the supplementary material available at *Biostatistics* online for sensitivity of the BF to such factors.

For comparison, Section S3 of the supplementary material available at *Biostatistics* online focuses on an analogous design, but one in which the sample size is fixed at 100 observations. In Figure S3 of the supplementary material available at *Biostatistics* online, the integrated risk and the operating characteristics are shown, providing overall a very similar picture to the one just described. In the same context, further insight into the behavior of the sampling prior average test error rates is obtained through visualization of the underlying conditional test error rates and truncated sampling prior distributions, shown in Figure S4 of the supplementary material available at *Biostatistics* online. Additionally, if it is required that specific targets for both testing and estimation are strictly controlled under the elicited sample size, then the goal sampling approach would be the method of choice. In Section S4 and Figure S5 of the supplementary material available at *Biostatistics* online, we thus illustrate the results of the sensitivity analyses for a goal sampling approach with target weighted sum of average error rates and AMSE equal to 0.027 and 0.006, respectively, i.e., the targets specified above to elicit the costs. Note that explicitly targeting alternative operating characteristics not included in the integrated risk definition may lead to sub-optimality of the sampling prior.

In Section S5 of the supplementary material available at *Biostatistics* online, we compare our results to the ones induced by the approach in which the test error costs do not include the prior normalizing constants, and thus the rejection threshold is defined as $\gamma^{\pi_a}=c'_0/(c'_0+c'_1)$, i.e., it is the same irrespective of the analysis prior specification. For the vague prior, the test decisions, and therefore the average test error rates of Figure S6 of the supplementary material available at *Biostatistics* online, are approximately equivalent to those in Figure 3, although weighted differently in the integrated risk. The remaining analysis priors tend to be *less* conservative than observed in Figure 3, although it is important to stress that this is induced by the fact that the trade-off between average type I and type II error rates is not optimized. The EB power prior and the mixture prior provide a compromise on the integrated risk, which is bounded and close to the one of the best performing analysis prior under each scenario.

Finally, Section S6 and Figures S7–S10 of the supplementary material available at *Biostatistics* online provide analogous comparisons for binomial outcomes. Key differences are introduced by the discreteness of the binomial distribution, which induces unsmooth declines of the operating characteristics for increasing sample sizes, and by the bounded parameter space, which leads, e.g., to a lower AMSE close to the boundaries.

## 5. Data application

We focus on the single-arm proof-of-concept trial with binomial outcome reported in Roychoudhury *and others* (2018). The trial was aimed at testing statistical significance and clinical relevance of an experimental drug for non-small-cell lung cancer and focused on objective response rate as a primary outcome. The threshold for significance $\theta_0$ was set at 0.075, while the threshold for relevance $\theta_R$ was identified as 0.175. The chosen analysis prior was a ${\rm Beta}(0.0811,1)$ (hereafter denoted RSN prior), which has mean equal to 0.075. The sample size of the trial was set at $n=25$, implying a conditional power at 0.275 equal to 0.858, which was regarded as acceptable. Following common practice, we assume that a relatively high confidence was therefore set on 0.275, and we assume a ${\rm Beta}(11,29)$ informative prior. We also assume that such prior is motivated by a historical study which observed $10$ successes and $28$ failures, and adopted a uniform prior specification. The EB power and the robust mixture priors are taken to be ${\rm Beta}(a_0 10+1,a_0 28+1)$, and $0.5 \cdot {\rm Beta}(11,29) + 0.5 \cdot {\rm Beta}(1,1)$, respectively.

**Fig. 4.**
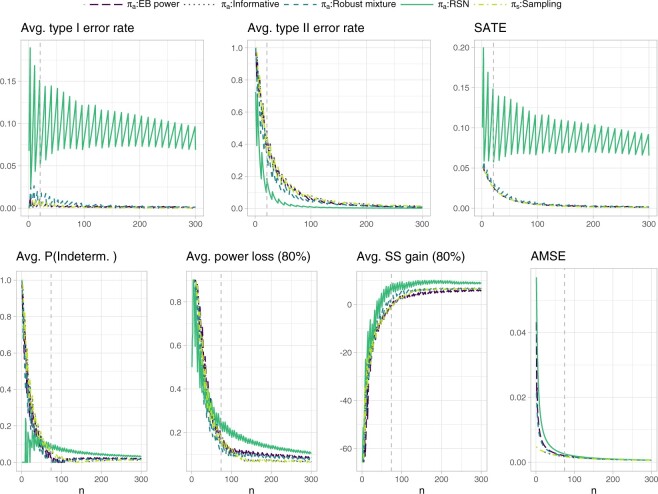
Operating characteristics related to testing and estimation for the single-arm proof-of-concept trial with binomial outcome reported in Roychoudhury *and others* (2018). The extreme power loss and extreme sample size gain in a subsequent Phase III trial, are defined as the 80% quantile of the respective distributions induced by the data outcomes. The probability of an indeterminate outcome, the extreme power loss and extreme sample size gain are averaged with respect to the sampling prior ${\rm Beta}(11,29)$ truncated from below at $\theta_R=0.175$. The remaining operating characteristics are averaged with respect to the whole sampling prior distribution. The dashed vertical lines represent the sample sizes identified for cost elicitation with respect to the RSN analysis prior, and, independently, for testing (upper panels, $n^{\rm SATE}=21$) and estimation (lower panels, $n^{\rm AMSE}=74$). Such sample sizes allow to maintain an average type I error rate and type II error rate below 0.15 and 0.2, respectively, as well as an average probability of an indeterminate outcome, average extreme power loss, and average extreme sample size gain below 0.1, 0.3, and 10, respectively. Note that the informative prior results exactly overlap with the sampling prior ones.

Figure 4 shows the operating characteristics related to testing and estimation. Focusing on the RSN prior, inspection of the weighted sum of average test error rates indicates that, for sample sizes larger than $n^{\rm SATE}=21$, the average type I error rate remains below 0.15 and the average type II error rate below 0.2. The weighted sum of average test error rates at $n^{\rm SATE}=21$ equals 0.058, and the derivative at this point (after local polynomial regression smoothing) gives approximately $c_n^{\rm SATE}= 2.04 \cdot 10^{-4}$. Focusing on the $AMSE$ target, we observed that, for sample sizes larger than $n^{\rm AMSE}=74$, the average probability of an indeterminate outcome remains below 0.1, the average extreme power loss ($1-\zeta=0.8$) below 0.3 and the average extreme sample size gain ($1-\zeta=0.8$) below 10. If this is deemed acceptable for $n^{\rm AMSE}=74$, then the target AMSE is $0.0026$, inducing $c^{\rm AMSE}_n=3.421 \cdot 10^{-5}$. The weight $w$ is then taken to be $n^{\rm SATE}/(n^{\rm SATE}+n^{\rm AMSE})=0.221$. The EB power and robust mixture prior are in this case much closer to the assumed sampling prior than the RSN prior, leading to an overall smaller weighted sum of average test error rates and AMSE.

**Fig. 5.**
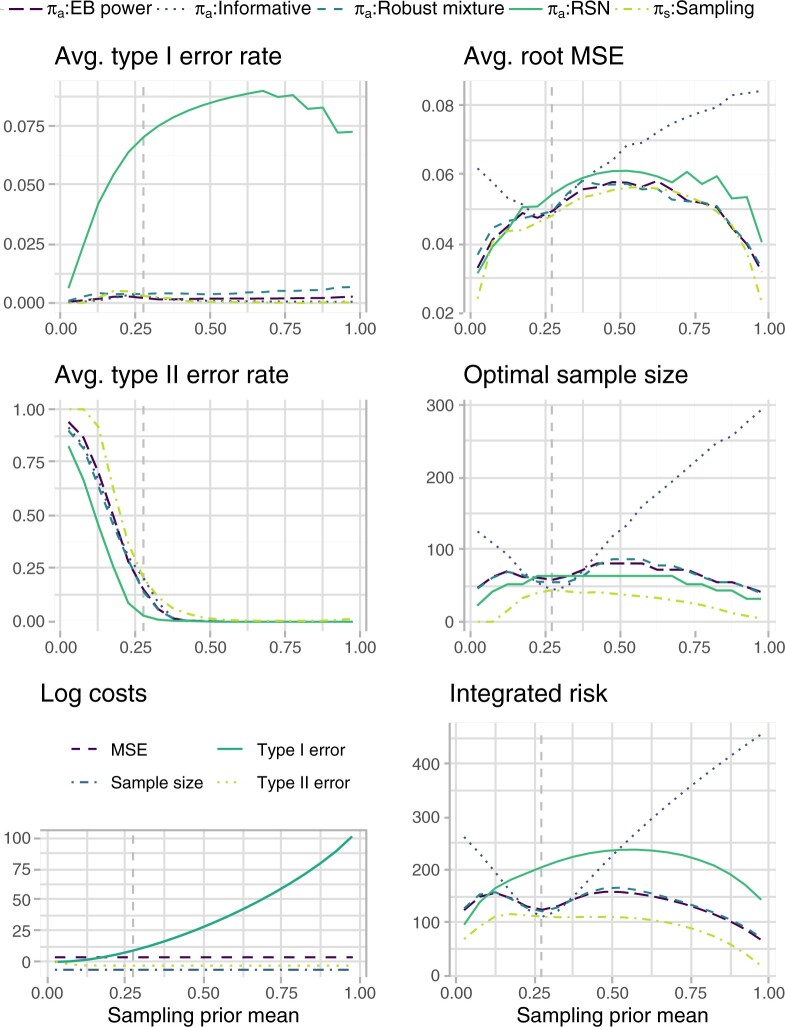
Integrated risk, costs, and operating characteristics for different analysis prior specifications and for varying sampling prior ${\rm Beta}(a_s,b_s)$ means $a_s/(a_s+b_s)$, $a_s+b_s=40$. The sample size is optimized to minimize the integrated risk. Costs are elicited as follows: $c_0=c_0' (1-P^{\pi}[\theta \leq \theta_0])^{-1}$, $c_1=c_1' (P^{\pi}[\theta \leq \theta_0])^{-1}$, $c_q=\{(1-w) c^{\rm SATE}_n\}/\{w c^{\rm AMSE}_n\},$ and $c_n = c^{\rm SATE}_n/w$ (see Equation (3.8)), where $c_0'=0.05$, $c_1'=0.95$, $c^{\rm SATE}_n=2.04 \cdot 10^{-4}$, $c^{\rm AMSE}_n=3.421 \cdot 10^{-5}$, and $w=0.221$. Note that test error log costs refer to the sampling prior specification. The dashed vertical line corresponds to the point at which the sampling prior and the historical information coincide, i.e., here we assume at 0.275 based on power calculations in Roychoudhury *and others* (2018).

Optimization of the integrated risk is then carried out with the selected costs, and sensitivity analyses are shown in Figure 5 for varying sampling prior ${\rm Beta}(a_s,b_s)$ means, under the constraint that $a_s+b_s=40$. The RSN analysis prior is, as expected, robust with respect to different sampling prior specifications, leading to sample sizes in the range $[22,64]$. The decrease near the boundaries is mainly induced by a lower sample size requirement for the MSE in these regions of the parameter space. Note that the sample size selected under the sampling prior with mean 0.275, 64, is relatively high if compared to the sample size originally elicited, 25. This increase is mostly induced by the estimation error component and, in turn, by the desire to limit the chance of incurring an extreme power loss in a Phase III study. The EB power and robust mixture prior show a better behavior, which is reflected in a globally lower integrated risk at the cost of a moderate increase in sample size; the only exception is for the region close to 0, where most of the RSN prior mass is concentrated.

## 6. Conclusions

In this work, we have explored different Bayesian procedures of sample size selection based on decision-theoretic considerations. In particular, we have defined an integrated risk which incorporates losses arising from testing, estimation, and sampling. We have related the loss arising from estimation to the probability of an indeterminate trial outcome as well as the potential power loss in a consequent Phase III trial, both aspects being of relevance in particular in the context of Phase II proof-of-concept trials. Furthermore, we have explored two possible elicitations of the test error costs, leading to test decisions based either on posterior probabilities or solely on BFs. The latter specification has the advantage of minimizing the weighted sum of the average type I and type II error rates, which may be appealing both in terms of optimization target and cost interpretability.

Skepticism about the historical information can be formally taken into account by performing sensitivity analyses based on the distinction between sampling and analysis prior. In particular, in the approach adopted here, decisions are based on the analysis prior, while uncertainty about $\theta$ is summarized by the sampling prior distribution. Frequentist operating characteristics are thus easily obtained by assuming a point-mass sampling prior.

We have illustrated the approach and compared different analysis prior specifications in a simulation example. Interestingly, in the set-up considered, when test decisions are based solely on the BF, the EB power prior induces test decisions which closely resemble the informative prior ones while retaining advantages in terms of AMSE robustness. The application of the methodology to a real proof-of-concept trial shows additionally that, if not only testing but also estimation is taken into account, particularly in view of a reduced potential for power loss in a subsequent Phase III trial, a higher Phase II sample size may be required.

We have focused on the commonly relevant targets in clinical trial design, i.e., test error rates and MSE, but of course alternative specifications of the loss function can be investigated (e.g., taking into account the distance of the true parameter value from $\theta_0$, see e.g., Lewis *and others*, 2007; Parmigiani and Inoue, 2009). Alternative specifications of the set of hypotheses, e.g., a simple null versus a composite alternative (see, e.g., Berger and Sellke, 1987, and references therein), as well as different, more complex, designs, can definitely be of interest for further research. A caveat in this direction is related to the dimensionality of the parameter space, due to the need to compute multiple integrals in the integrated risk, and to the availability of analytical forms for the posterior, due to the need to otherwise resort to, e.g., Markov Chain Monte Carlo.

One general criticism of decision-theoretic approaches in the clinical trial context is that different interests may be at stake, inducing a lack of consensus about the costs and losses (see Spiegelhalter *and others*, 2004, and references therein). We have outlined a “simplified” approach to cost elicitation, linked to the implicit cost elicitation of “goal sampling” approaches. However, we stress that actual costs of test and estimation errors should be adopted whenever available. An interesting proposal moving in the direction of a more objective cost elicitation is provided in Isakov *and others* (2019), where the definition of type I and type II error costs is based on disease prevalence, severity, and cost of adverse events. The authors also follow a decision-theoretic approach for optimal sample size elicitation; one key difference from our work is the focus on a two-point mass prior and on conditional error rates at each of these two points.

We believe that our study can be of particular interest in view of an increased need for rationales of type I error rate inflation when incorporation of historical information is desired. Moreover, both a test decision based on the principle of comparing evidence in favor of the alternative versus the null hypothesis, as a BF thresholding implies, and the monitoring of a relevance threshold, move in the direction of abandoning a purely significance target. More broadly, interest in alternatives to $p$-values and significance testing is now on the rise, as shown by the *ASA statement on $p$-values and Statistical Significance* (Wasserstein and Lazar, 2016), and the recent issue of the American Statistician “Statistical Inference in the 21st Century: A World Beyond $p < 0.05.$” The article by Ruberg *and others* (2019) contained in this issue indeed supports a broader use of the Bayesian paradigm and of relevant external information in drug development and approval. A formal decision-theoretic approach additionally allows adopting test and estimation decisions which reflect an explicit elicitation of the targets to be optimized, and depend on the *“actual costs, benefits, and probabilities”* (Gelman and Robert, 2014).

## 7. Software

R code for reproduction of all simulations and figures is available online at https://github.com/BDTTrialDesigns/BDT_CombIRSens.

## Supplementary Material

kxaa027_Supplementary_Data
