# Supplementary material for: A decision-theoretic approach to Bayesian clinical trial design and evaluation of robustness to prior-data conflict
Source: Biostatistics. 2020 Jul 31;23(1):328–44. doi: 10.1093/biostatistics/kxaa027 (PMC9118338; doi:10.1093/biostatistics/kxaa027)
Supplement: kxaa027_Supplementary_Data [file kxaa027_supplementary_data.zip › kxaa027-suppl_data/biosts-19235-File002.pdf]

Supplementary Material for

A decision-theoretic approach to Bayesian clinical trial design and robustness to prior-data conflict

Silvia Calderazzo, Manuel Wiesenfarth, Annette Kopp-Schneider

S1 Cost elicitation and interpretation

Here we graphically explore the procedure for cost elicitation outlined in Section 3.2 of the main text. Recall that to reach a target weighted sum of average test error rates, $SATE$, a minimum sample size n^{SATE} is required, where larger sample sizes correspond to more ambitious targets. The cost per observation corresponding to this combination can be inferred noting that $r^{SATE}(n) = SATE(n) + c_n^{SATE} n$ is minimised if $dr^{SATE}(n)/dn = 0$, i.e. if $c_n^{SATE} = -\frac{dSATE(n)}{dn}|_{n^{SATE}}$. This is a local approximation in the sense that the cost would effectively vary with varying $SATE$ targets, following the rationale that a lower $SATE$ target implies the 'readiness' to sacrifice a smaller number of samples and therefore an increased cost per sample. The same procedure can be applied to the average MSE target, $AMSE$. An illustration of the weighted sum of average test error rates and average MSE targets decrease for increasing sample size and of the corresponding negative derivatives is provided in Supplementary Figure S1. Here we focus on a normal outcome $\mathbf{y}$, $y_i \sim N(\theta, \sigma^2 = 1)$, $i = 1, \ldots, n$, assuming a vague analysis prior specification $N(0, 10^2)$, and sampling prior $N(0.25, 1/50)$, as in Section 4 of the main text.

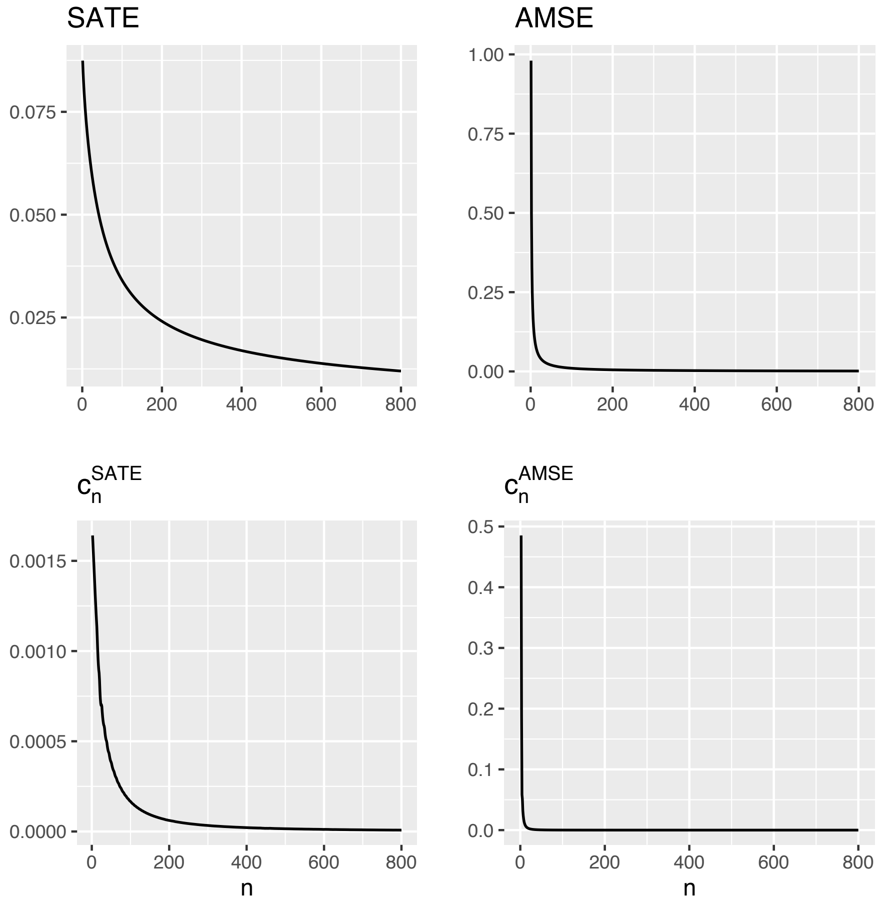

Figure S1: Weighted sum of average test error rates and average MSE (top panels), for increasing sample size n of normal observations with unit variance. The values are computed for decisions based on a vague prior specification $N(0, 10^2)$, and averaged with respect to a $N(0.25, 1/50)$ sampling prior. Approximate negative derivatives, giving rise to the costs, are shown in the corresponding bottom panels.

S2 Bayes factor sensitivity

Here we illustrate the impact of the historical information location, and of the weights and vague prior component specifications for the robust mixture prior, on the Bayes factor. Figure S2 shows the BF behaviour when the historical data mean $\bar{y}_0$ equals 0.25, as in the simulation example of Section 4 in the main text, or $\bar{y}_0 = 0.5$, i.e. the alternative hypothesis is *a priori* more strongly supported. Moreover, we consider two alternative specifications of the $0.5 \cdot N(\bar{y}_0, 1/50) + 0.5 \cdot N(\bar{y}_0, 10^2)$ prior, referred to as 'robust mixture', elicited for the simulation study: a robust mixture prior with unit information vague component ('robust mixture (unit information)', see [1] : $0.5 \cdot N(\bar{y}_0, 1/50) + 0.5 \cdot N(\bar{y}_0, 1)$), and a robust mixture prior with increased weight for the informative component ('robust mixture (0.8)': $0.8 \cdot N(\bar{y}_0, 1/50) + 0.2 \cdot N(\bar{y}_0, 10^2)$).

Focusing first on $\bar{y}_0 = 0.25$, we observe, as already noted in the main text, almost overlapping EB power and informative prior BF curves in the region of interest for the test decisions. The 'robust mixture' prior behaves more similarly to the vague prior specification: such similarity becomes even stronger when a unit information vague component is adopted. The similarity also increases when more weight is assigned to the informative component: this is a consequence of the robust mixture prior resembling more closely the informative prior. Note that, while the EB power prior effectively varies with data outcomes, the robust mixture prior remains fixed, and thus the likelihood is always averaged with respect to the same 'truncated' prior distributions. As the prior odds are not accounted for in the test decision, the shape of the prior is the key factor influencing the BF behaviour. This is even more evident for $\bar{y}_0 = 0.5$, where we notice the EB power prior BF approaching the informative prior BF as we get closer to 0.5, while it becomes closer to the vague prior BF as increasing evidence of conflict is observed. The 'robust mixture' and 'robust mixture (0.8)' tend to behave almost identically in the region of interest for the test decision, while the 'robust mixture (unit information)' tends to reach BF values between the vague and informative/EB power prior ones.

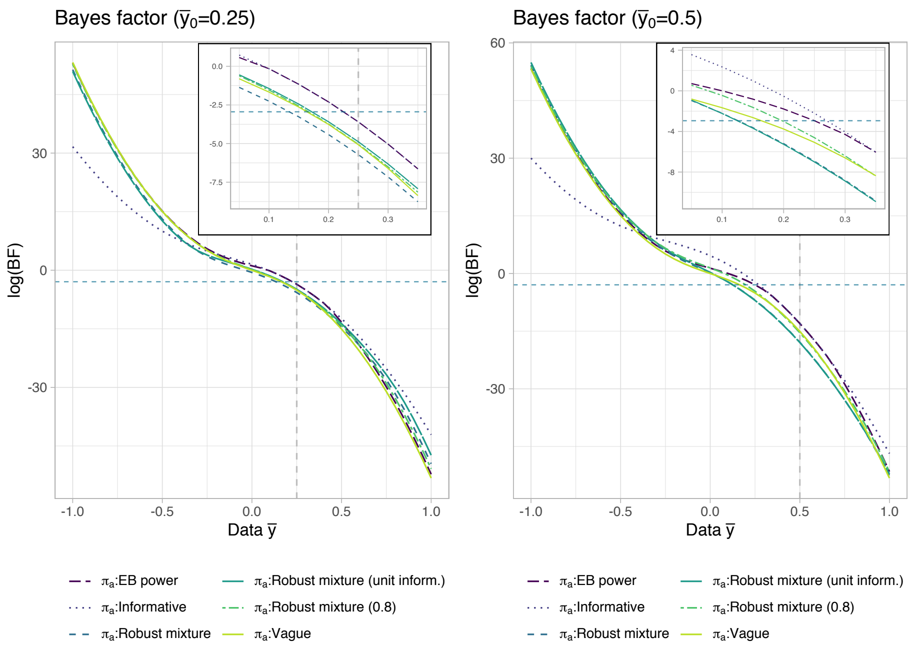

Figure S2: Bayes factor, on the logarithmic scale, for analysis priors assuming the location of historical information equal to 0.25 (left), and 0.5 (right), and for varying current data mean outcomes. The sample size is assumed equal to 100 in both plots. The dashed horizontal line corresponds to $\log(c_0'/c_1')$, $c_0' = 0.05$ and $c_1' = 0.95$, i.e. the log- (Bayes factor) threshold for rejection, see Equation (2.6) in the main text. Priors are defined as in Section 4 of the main text, with the addition of the 'unit inform.' and '80' robust mixture prior specifications corresponding, respectively, to a $0.5 \cdot N(\bar{y}_0, 1/50) + 0.5 \cdot N(\bar{y}_0, 1)$ and a $0.8 \cdot N(\bar{y}_0, 1/50) + 0.2 \cdot N(\bar{y}_0, 10^2)$ density.

S3 Normal outcome, costs incorporating prior normalising constants, and fixed sample size

In this section we focus on the same design described in Section 4 of the main text, but the sample size is fixed at 100 observations. Supplementary Figure S3 shows the overall behaviour of the integrated risk and of the operating characteristics. The most evident difference with respect to the scenario in which sample size is optimised, can be identified in the vague and sampling prior average MSE values, which remain constant across different sampling prior mean values, as expected.

It is worth noting that the sampling prior results are somewhat artificial, i.e. the sampling prior is effectively changing as we move along μ_s, and thus are only aimed at representing the best achievable result if we could exactly guess our informative analysis prior. To gain further insight into the irregular behaviour of its average type I error rate, we show in Supplementary Figure S4 a sample subset of the truncated sampling priors and conditional error rates computed under the same cost specification as Supplementary Figure S3. In the top panel, we can observe that both type I and type II conditional error rates decrease to zero when moving away from $\theta_0 = 0$, for all sampling priors; this is sensible as it is easier to discriminate between the two hypotheses. When the (truncated) sampling prior places significant mass on such extreme values, the corresponding average test error rates are also close to zero. As we approach zero, conditional type I error rates are larger for sampling priors with a negative mean, and smaller for sampling priors with positive means, and vice-versa for type II conditional error rates (although the latter achieves higher values, due to the imbalance in the costs). This behaviour is induced by the fact that outcomes close to zero have, under both hypotheses, a relatively high likelihood, which is, however, weighted very heavily in the Bayes factor by the (truncated portion of the) sampling prior with a spike at zero (see bottom panel). This leads also to an increase in average test error rates, which reaches its maximum when relatively high conditional test error rates also receive a significant weight in the average with respect to the (truncated) sampling prior, e.g. when $\mu_s = -0.35$.

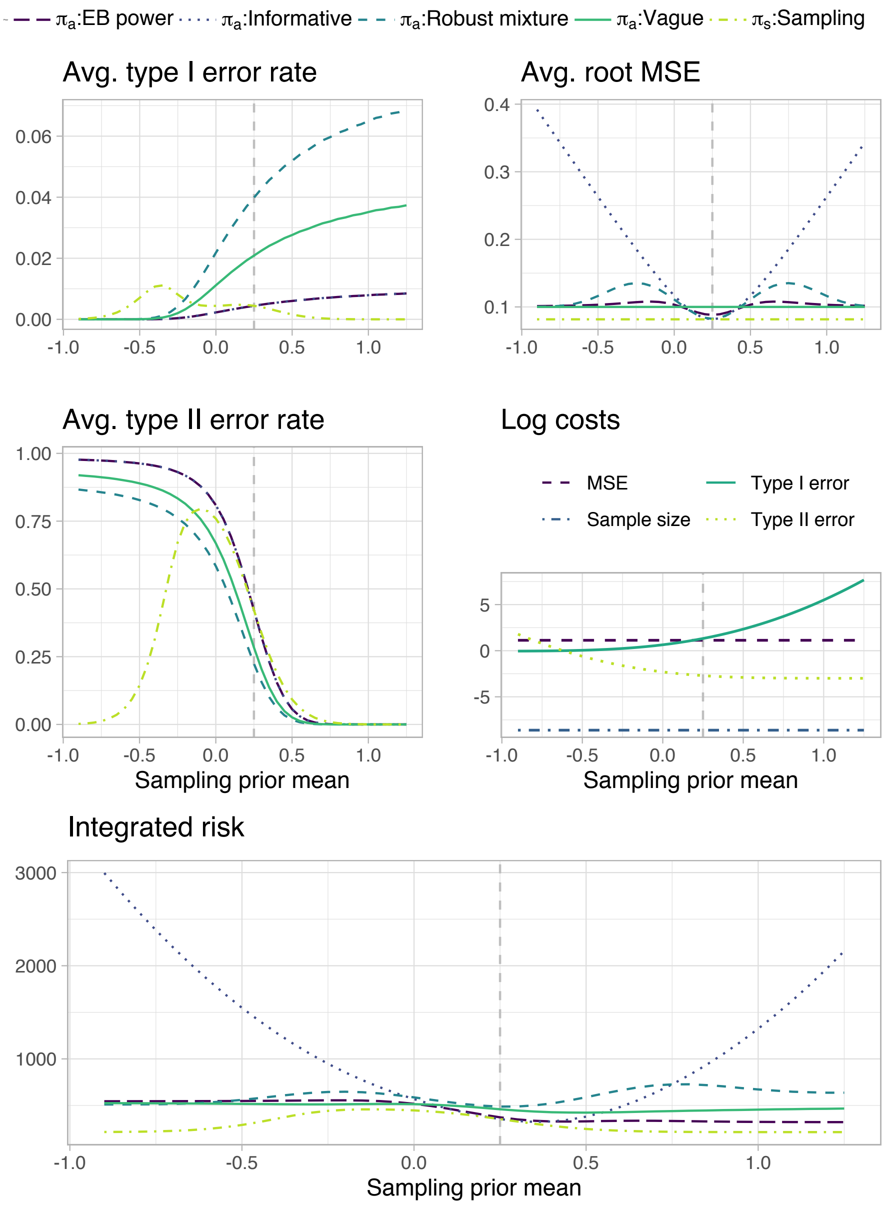

Figure S3: Integrated risk, costs, and operating characteristics for different analysis prior specifications and for varying sampling prior means μ_s. The sampling prior variance is fixed at $\sigma_s^2 = 1/50$, and the sample size is fixed at 100. Costs are elicited as in Figure 3 of the main text, i.e.: $c_0 = c_0'(1 - P^\pi[\theta \leq \theta_0])^{-1}$, $c_1 = c_1'(P^\pi[\theta \leq \theta_0])^{-1}$, $c_q = \{(1-w)c_n^{SATE}\}/\{wc_n^{AMSE}\}$ and $c_n = c_n^{SATE}/w$ (see Equation (3.8) in the main text), where $c_0' = 0.05$, $c_1' = 0.95$, $c_n^{SATE} = 8.511 \cdot 10^{-5}$, $c_n^{AMSE} = 3.098 \cdot 10^{-5}$ and $w = 0.471$. Note that test error log costs refer to the sampling prior specification. The dashed vertical line corresponds to the point at which the sampling prior and the historical information coincide.

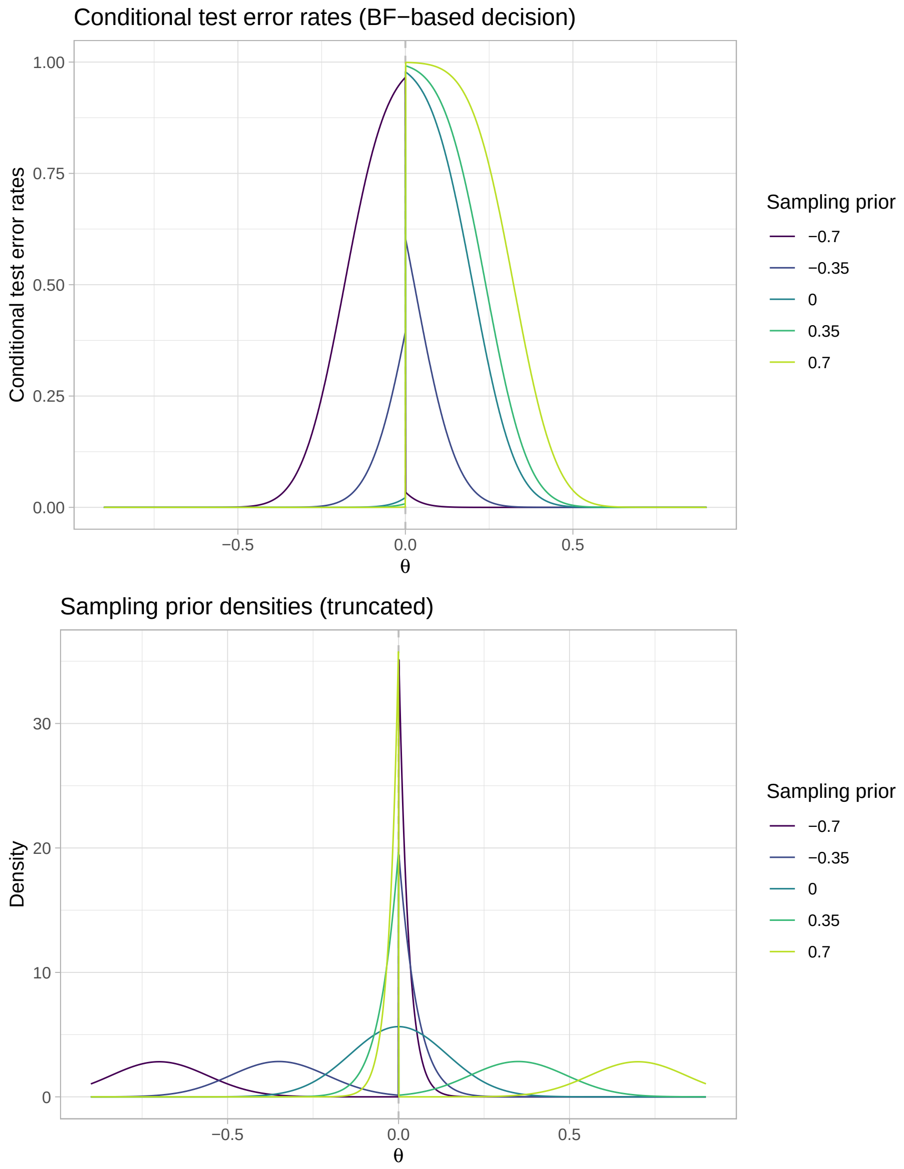

Figure S4: Conditional type I and type II error rates (top) and truncated sampling priors (bottom) for the same set-up as Supplementary Figure S3. Results are shown for five different exemplary sampling priors, with varying sampling prior mean μ_s. The sampling prior variance is fixed at $\sigma_s^2 = 1/50$. The test decision is based on comparison of the Bayes factor with the ratio c_0'/c_1', $c_0' = 0.05$ and $c_1' = 0.95$, (corresponding to test error costs: $c_0 = c_0'(1 - P^{\pi_s}[\theta \leq \theta_0])^{-1}$, $c_1 = c_1'(P^{\pi_s}[\theta \leq \theta_0])^{-1}$).

S4 Goal sampling

We now turn to a scenario in which the optimal sample size is selected as the minimum to reach specific testing and estimation targets. Here we focus on a scenario in which the target weighted sum of average test error rates is equal to 0.027 and the target average MSE is equal to 0.006. Such values are chosen according to the cost elicitation described in Section 4 in the main text. Supplementary Figure S5 shows the minimum required sample size and the resulting operating characteristics of the different analysis priors, and, for comparison, of the sampling prior. Overall, we observe that for moderately positive values of μ_s, the average MSE target tends to dominate in the choice of the sample size, with the exception of the robust mixture prior specification, while the reverse is observed for negative values of μ_s, where the target sum of average error rates represents a quite ambitious target which induces optimal sample sizes above the maximum allowed for all of the analysis priors.

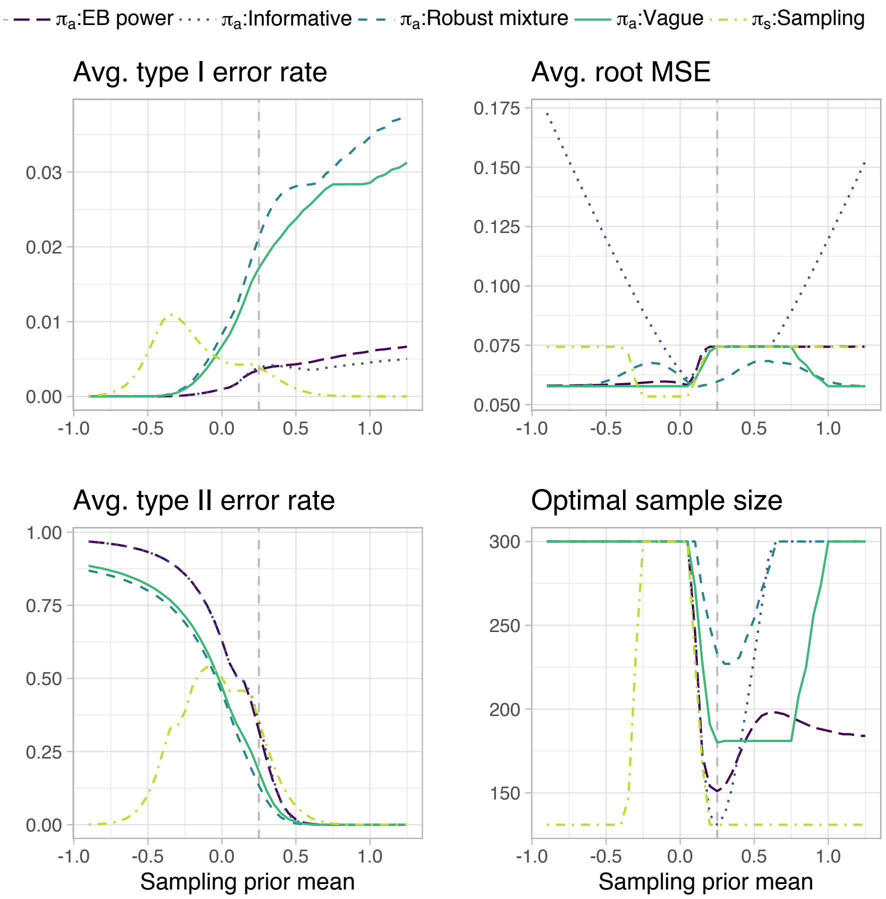

Figure S5: Operating characteristics and minimum required sample size for varying sampling prior means μ_s and fixed sampling prior variance $\sigma_s^2 = 1/50$. Costs are elicited as follows: $c_0 = c_0'(1 - P^\pi[\theta \leq \theta_0])^{-1}$, $c_1 = c_1'(P^\pi[\theta \leq \theta_0])^{-1}$, $c_0' = 0.05$ and $c_1' = 0.95$. The target weighted sum of average test error rates is equal to 0.027 and the target average MSE to 0.006. The dashed vertical line corresponds to the point at which the sampling prior and the historical information coincide. The maximum sample size is truncated at n=300.

S5 Normal outcome, fixed costs, and optimised sample size

In this section we focus on a design in which the test error costs do not include the prior normalising constants, and thus the rejection threshold is fixed to $\gamma^{\pi_a} = c'_0/(c'_0 + c'_1)$ for all analysis priors.

In Supplementary Figure S6 we observe that, as the test decision does incorporate the prior odds, the average type I and type II error rates of the sampling prior are generally increasing and decreasing, respectively, as we increase μ_s. The test decisions are now also influenced by the prior odds of each analysis prior and this leads to a much closer behaviour of the EB power and robust mixture priors, which is also reflected in terms of optimality of the integrated risk. A more asymmetric behaviour than in Figure 3 of the main text in terms of integrated risk, and larger oscillations in terms of optimal required sample size, can be observed for the EB power prior. Note that, to enhance comparability, we have retained the same cost specifications for c_q and c_n as in Figure 3 of the main text.

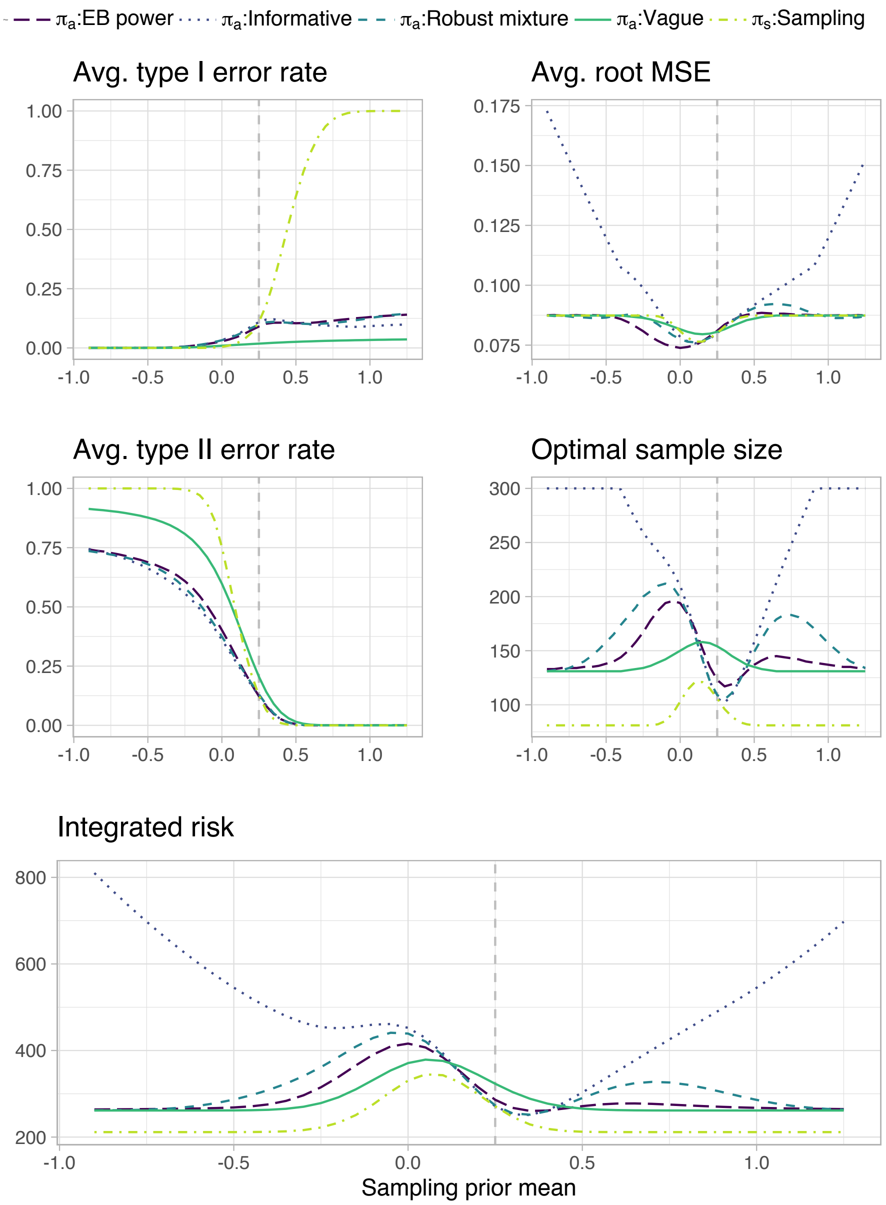

Figure S6: Integrated risk, costs, and operating characteristics for different analysis prior specifications and for varying sampling prior means μ_s. The sampling prior variance is fixed at $\sigma_s^2 = 1/50$, and the sample size is optimised to minimise the integrated risk. Costs are elicited as in Figure 3 of the main text, i.e.: $c_0 = c_0' = 0.05$, $c_1 = c_1' = 0.95$, $c_q = \{(1-w)c_n^{SATE}\}/\{wc_n^{AMSE}\}$ and $c_n = c_n^{SATE}/w$ (see Equation (3.8) in the main text), where $c_n^{SATE} = 8.511 \cdot 10^{-5}$, $c_n^{AMSE} = 3.098 \cdot 10^{-5}$ and $w = 0.471$. The dashed vertical line corresponds to the point at which the sampling prior and the historical information coincide. The maximum sample size is truncated at n=300.

S6 Binomial outcome

We focus on a binomial outcome $\mathbf{y}$, $y_i \sim Ber(\theta)$, $i = 1, \dots, n$. Interest is placed on the proportion θ, which is assumed to follow a $Beta(a_s, b_s)$ sampling prior. We test the set of hypotheses $H_0 : \theta \leq 0.2$ versus $H_1 : \theta > 0.2$ with a relevance threshold $\theta_R = 0.4$. In analogy to the normal outcome case, we consider as analysis priors: (i) a uniform prior, which follows a $Beta(1, 1)$ distribution; (ii) an informative prior, which follows a $Beta(25, 25)$ distribution; (iii) a robust mixture prior, which is a mixture of the informative prior and the vague prior distribution, with mixture weight equal to 0.5; and (iv) an empirical Bayes (EB) power prior π_{pow}, $Beta(a_0 24 + 1, a_0 24 + 1)$, which can be interpreted as the posterior of a trial adopting a uniform prior specification and collecting $a_0 24$ successes, and $a_0 24$ failures.

We retain $c_0' = 0.05$ and $c_1' = 0.95$. Again, we first focus on a choice of the test error costs which does include the prior normalising constants. Visual inspection of the operating characteristics can be carried out based on Figure S7, assuming that the sampling prior coincides with the informative analysis prior. Owing to the discreteness of the binomial outcome, we observe generally unsmooth declines of the operating characteristics.

As in the normal outcome case in the main text, we focus on the vague (uniform) analysis prior to elicit our costs. We aim to reach an average type II error rate below 0.2 (the average type I error rate is below 0.05 for any sample size), thus we can hypothesise readiness to sample $n^{SATE} = 25$ samples for the testing target, leading to a weighted sum of average error rates equal to 0.020. As for estimation, $n^{AMSE} = 64$ is required to reach an average probability of an indeterminate outcome under $\theta > \theta_R$ below 0.1 and an average extreme power loss (we take the $1 - \zeta = 0.8$ quantile of the data induced distribution) and average extreme sample size gain (again for $1 - \zeta = 0.8$) below 0.3 and approximately 5, respectively. The average MSE at $n^{AMSE} = 64$ is approximately 0.0036. We then obtain $w = 0.281$, $c_n^{SATE} = 5.288 \cdot 10^{-4}$, and $c_n^{AMSE} = 5.378 \cdot 10^{-5}$. Note that computation of the costs is carried out after smoothing of the weighted sum of average test error rates and average MSE curves via local polynomial regression (function `loess` the R `stats` package).

We perform a sensitivity analysis of the integrated risk and of the optimal sample size by varying a_s and b_s from 1 to 49, under the constraint $a_s + b_s = 50$, i.e. the sample size of the historical trial is kept fixed. Figure S8 illustrates the result. Note that all analysis priors tend to behave quite similarly in terms of testing and estimation, with the exception of the informative prior, which shows clear unrobustness as the sampling prior mean deviates from the location of the historical information. The robust mixture and EB power prior also achieve some relative advantages with respect to the vague prior in the region where historical information and the sampling prior are consistent, as expected. Average type I error rates are unfortunately slightly noisy, owing to the small values and the discreteness of the binomial, and thus comparisons are more challenging. In analogy to the normal outcome scenario, costs for each test error based on the sampling prior specification tend to increase in opposite directions

as the sampling prior increasingly favours the null or alternative hypothesis.

In analogy to the normal outcome study, we additionally compute the operating characteristics in the case where the test error costs do not incorporate the prior normalising constants, and for a 'goal sampling' approach. Supplementary Figure S9 displays the operating characteristics and integrated risk with fixed test error costs, and, in contrast with the normal outcome example, we can most notably observe that the average type I error rate of the informative prior increases much more steeply; this is consistent with the behaviour of the sampling prior, which suggests that the role of historical information is much more predominant in ruling out the null hypothesis. However, we observe optimal sample sizes for all analysis priors very close to those of the approach which includes the prior normalising constants in the costs, suggesting that the average MSE components play the predominant role in sample size elicitation. The goal sampling approach with costs incorporating the prior normalising constants is shown in Figure S10, which suggests again that the target weighted sum of average error rates elicited when the sampling prior has mean 0.275 ($SATE = 0.020$) represents a quite ambitious target for all the analysis priors considered as we move towards 0; on the other hand, as we move above the relevance threshold and towards 1, the average MSE target ($AMSE = 0.0036$) tends to dominate the sample size requirement.

References

[1] Robert E Kass and Larry Wasserman. The selection of prior distributions by formal rules. *Journal of the American Statistical Association*, 91(435):1343–1370, 1996.

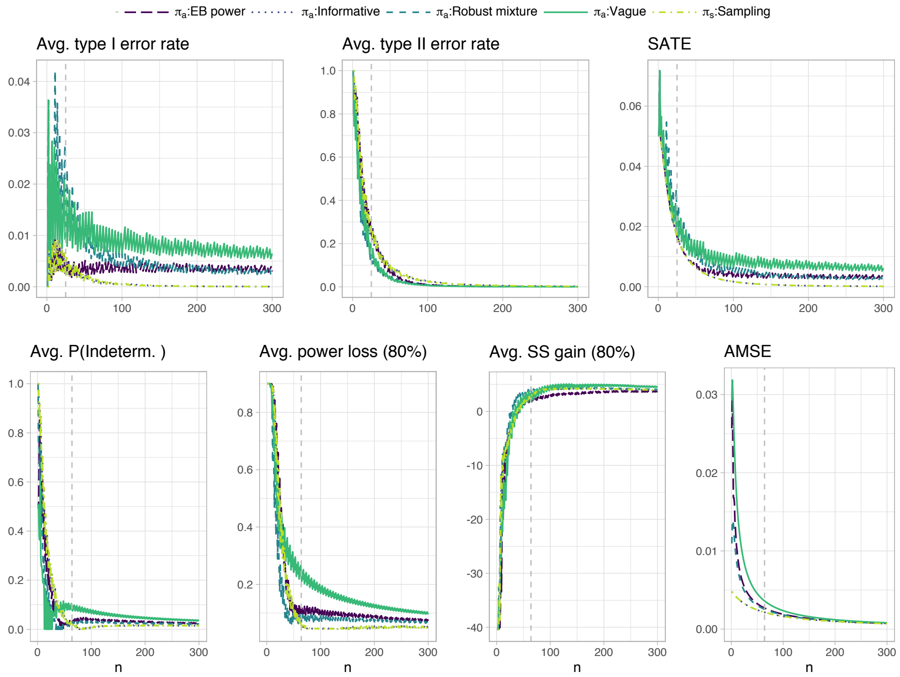

Figure S7: Operating characteristics related to testing and estimation for the binomial outcome simulation example. The extreme power loss and extreme sample size gain in a subsequent Phase III trial, are defined as the 80% quantile of the respective distributions induced by the data outcomes. The probability of an indeterminate outcome, the extreme power loss and extreme sample size gain are averaged with respect to the sampling prior $Beta(25, 25)$ truncated from below at $\theta_R = 0.4$. The remaining operating characteristics are averaged with respect to the whole sampling prior distribution. The dashed vertical lines represent the sample sizes identified for cost elicitation with respect to the vague (uniform) analysis prior, and, independently, for testing (upper panels, $n^{SATE} = 25$) and estimation (lower panels, $n^{AMSE} = 64$). Such sample sizes allow maintaining an average type II error rate below 0.2 (average type I error rate is below 0.05 for all sample sizes), as well as an average probability of an indeterminate outcome, average extreme power loss and average extreme sample size gain below 0.1, 0.3, and approximately 5, respectively. Note that the informative prior results exactly overlap with the sampling prior ones.

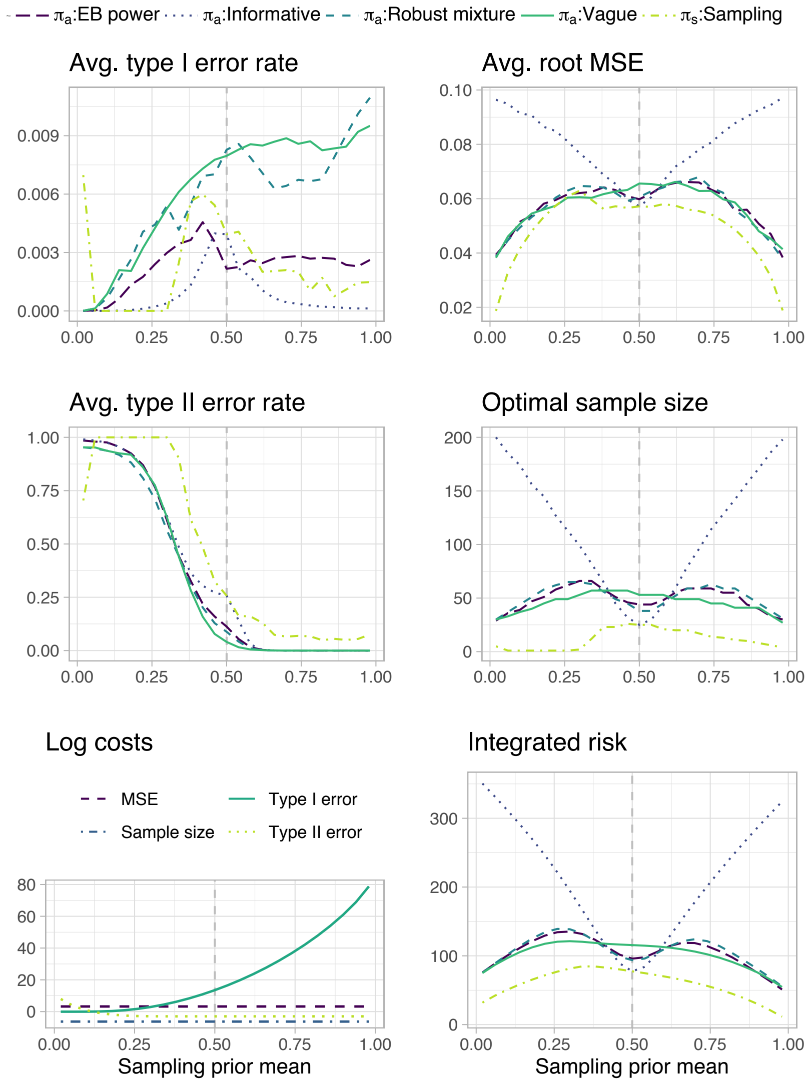

Figure S8: Integrated risk, costs, and operating characteristics for different analysis prior specifications and for varying sampling prior means $a_s/(a_s + b_s)$, $a_s + b_s = 50$. The sample size is optimised to minimise the integrated risk. Costs are elicited as follows: $c_0 = c_0'(1 - P^\pi[\theta \leq \theta_0])^{-1}$, $c_1 = c_1'(P^\pi[\theta \leq \theta_0])^{-1}$, $c_q = \{(1-w)c_n^{SATE}\}/\{wc_n^{AMSE}\}$ and $c_n = c_n^{SATE}/w$ (see Equation (3.8) in the main text), where $c_0' = 0.05$, $c_1' = 0.95$, $c_n^{SATE} = 5.288 \cdot 10^{-4}$ and $c_n^{AMSE} = 5.378 \cdot 10^{-5}$ and $w = 0.281$. Note that test error log costs refer to the sampling prior specification. The dashed vertical line corresponds to the point at which the sampling prior and the historical information coincide

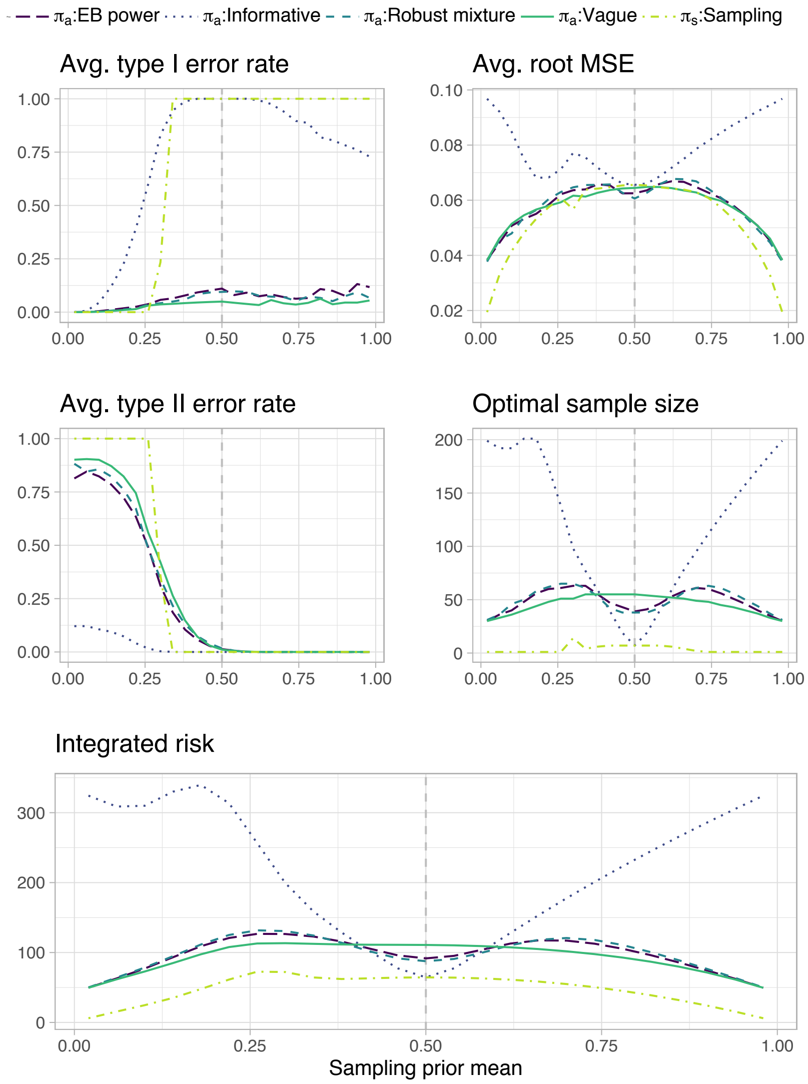

Figure S9: Integrated risk, costs, and operating characteristics for different analysis prior specifications and for varying sampling prior means $a_s/(a_s + b_s)$, $a_s + b_s = 50$. The sample size is optimised to minimise the integrated risk. Costs are elicited as follows: $c_0 = c'_0 = 0.05$, $c_1 = c'_1 = 0.95$, $c_q = \{(1 - w)c_n^{SATE}\}/\{wc_n^{AMSE}\}$ and $c_n = c_n^{SATE}/w$, $c_n^{SATE} = 5.288 \cdot 10^{-4}$, $c_n^{MSE} = 5.378 \cdot 10^{-5}$ and $w = 0.281$. The unsmooth behaviour of the average type I error rate is due to its small values and the discreteness of the binomial. The dashed vertical line corresponds to the point at which the sampling prior mean and the mean derived from the historical information coincide.

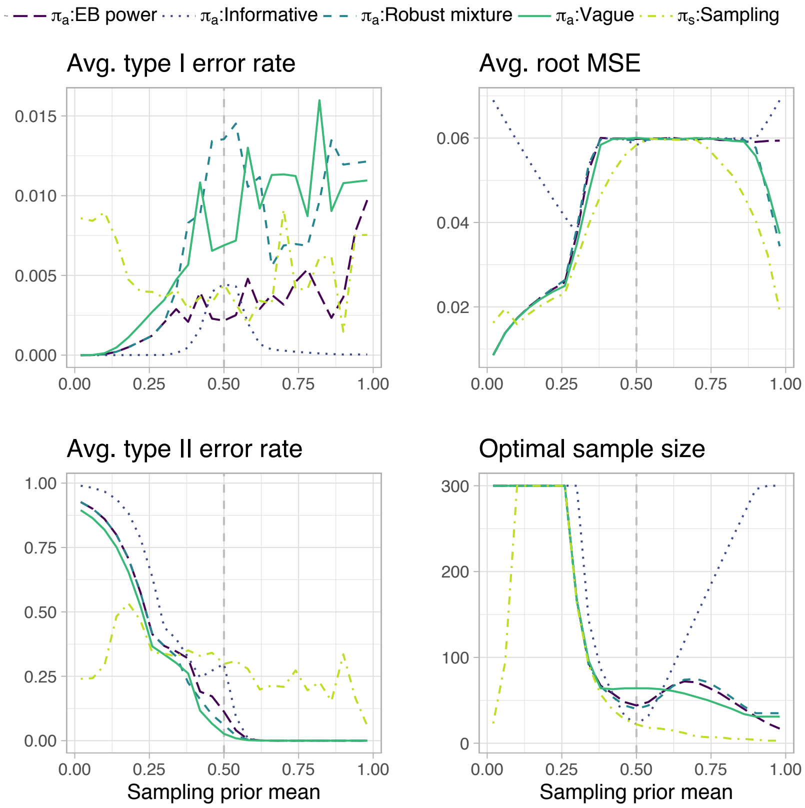

Figure S10: Operating characteristics and minimum required sample size and for varying sampling prior means $a_s/(a_s + b_s)$, $a_s + b_s = 50$. Costs are elicited as follows: $c_0 = c_0'(1 - P^\pi[\theta \leq \theta_0])^{-1}$, $c_1 = c_1'(P^\pi[\theta \leq \theta_0])^{-1}$, $c_0' = 0.05$ and $c_1' = 0.95$. In analogy with the cost elicitation process, the target weighted sum of average test error rates is equal to 0.020 and the target average MSE to 0.0036. The unsmooth behaviour of the average type I error rate is due to its small values and the discreteness of the binomial. The dashed vertical line corresponds to the point at which the sampling prior and the historical information coincide. The maximum sample size is truncated at n=300.
